# Association of Neurodevelopmental Outcomes With Environmental Exposure to Cyclohexanone During Neonatal Congenital Cardiac Operations: A Secondary Analysis of a Randomized Clinical Trial

**DOI:** 10.1001/jamanetworkopen.2020.4070

**Published:** 2020-05-06

**Authors:** Allen D. Everett, Jessie P. Buckley, Greg Ellis, Jun Yang, David Graham, Megan Griffiths, Melania Bembea, Eric M. Graham

**Affiliations:** 1 Division of Pediatric Cardiology, Department of Pediatrics, Johns Hopkins University, Baltimore, Maryland; 2 Johns Hopkins Bloomberg School of Public Health, Department of Environmental Health and Engineering, Johns Hopkins University, Baltimore, Maryland; 3 Department of Anesthesia and Critical Care Medicine, Johns Hopkins University, Baltimore, Maryland; 4 Molecular Determinants Core, Johns Hopkins All Children’s Hospital, St Petersburg, Florida; 5 Department of Pediatrics, Division of Cardiology, Medical University of South Carolina; Charleston

## Abstract

**Importance:**

Cyclohexanone is an industrial solvent used as a coupling agent in medical plastics. Perioperative exposure to cyclohexanone could play a role in lower scores on measures of neurodevelopmental outcomes after neonatal cardiac operations.

**Objective:**

To examine the presence and association of serum cyclohexanone level with neonatal cardiac operations and neurodevelopmental outcomes.

**Design, Setting, and Participants:**

This ad hoc secondary analysis used data from the Corticosteroid Therapy in Neonates Undergoing Cardiopulmonary Bypass randomized clinical trial. The cohort included neonates younger than 31 days and with at least 37 weeks postgestational age at surgical treatment who were enrolled at a single center between June 1, 2012, and October 31, 2016, and who had completed a neurodevelopmental assessment at age 12 months. Data were analyzed from July 8 to August 20, 2019.

**Exposures:**

Serum cyclohexanone and its metabolites were measured preoperatively (prior to skin incision), postoperatively (immediately after the surgical procedure was completed), and 12 hours postoperatively. Cyclohexanone and the molar sum of its metabolites were examined at each point and as a geometric mean of all 3 points.

**Main Outcomes and Measures:**

Neurodevelopment was assessed at age 12 months with the Bayley Scales of Infant and Toddler Development III, assessing cognitive, language, and motor function composite scores standardized to a population mean (SD) of 100 (15). Linear regression models were used to determine covariate-adjusted differences in 12-month cognitive, language, and motor composite scores per interquartile range increase in cyclohexanone level or summed metabolite molar concentrations.

**Results:**

Among 85 included neonates, mean (SD) age at surgical treatment was 9.7 (5.3) days, 49 (58%) were boys, and 54 (64%) underwent corrective repair. Mean (SD) Bayley Scales of Infant and Toddler Development III composite scores were 108.2 (12.2) for cognitive function, 104.7 (11.0) for language function, and 94.7 (15.7) for motor function. Median (interquartile range) cyclohexanone levels increased approximately 3-fold from immediately prior to surgical treatment to immediately after surgical treatment (572 [389-974] vs 1744 [1469-2291] μg/L; *P* = .001). In adjusted analyses, higher geometric mean cyclohexanone levels were associated with significantly lower composite scores for cognitive (−4.23; 95% CI, −7.39 to −1.06; *P* = .01) and language (−3.65; 95% CI, −6.41 to −0.88; *P* = .01) function. The difference in composite scores for motor function among infants with higher geometric mean cyclohexanone levels was not statistically significant(−3.93, 95% CI: −8.19 to 0.33, *P* = .07).

**Conclusions and Relevance:**

The findings of this secondary analysis of a randomized clinical trial suggest that infants who underwent neonatal cardiac surgical treatment with cardiopulmonary bypass had substantial cyclohexanone levels, which were associated with adverse neurodevelopmental function at age 12 months.

**Trial Registration:**

ClinicalTrials.gov identifier: NCT01579513

## Key Points

**Question:**

Are neonatal heart operations associated with cyclohexanone exposure and neurodevelopmental outcomes at age 12 months?

**Findings:**

In this secondary analysis of a randomized clinical trial of neonatal cardiac operations with cardiopulmonary bypass that included 85 neonates, neonates had a 3-fold increase in serum cyclohexanone levels after cardiopulmonary bypass. Increased geometric means of serum cyclohexanone levels were independently associated with lower composite scores for cognitive and language functions.

**Meaning:**

These findings suggest that neonatal cardiac surgery with cardiopulmonary bypass was associated with substantial cyclohexanone exposure, which was independently associated with adverse neurodevelopment at age 12 months.

## Teaser

This secondary analysis of a randomized clinical trial examines serum concentration of cyclohexanone in neonates undergoing surgical treatment for congenital cardiac disease and the association of this cyclohexanone exposure with cognitive, language, and motor functions at age 12 months.

## Introduction

Neurodevelopmental delays and disabilities are the most frequent and significant consequences of congenital heart disease (CHD) and its treatment.^1^ Improvements in surgical techniques and medical support have reduced mortality and morbidity rates, including overt neurological injury (eg, seizure, coma, death) from 50% to 2.3%.^2,3^ However, sensitive magnetic resonance imaging studies have identified clinically silent, new brain injuries as small focal white matter lesions or strokes in more than 60% of newborns who undergo surgical treatment.^4,5^ Consistent with the focal nature of these injuries, cognitive deficits at school age are mild, with mean IQs reported in the low end of the reference range.^6^ However, children with CHD exhibit deficits in multiple neurodevelopmental domains, including speech and language, attention, memory, executive function, visual-spatial skills, and gross and fine motor function, suggesting a more global injury not resolved by magnetic resonance imaging. This high prevalence of low-severity anomalies has been termed the *neurodevelopmental signature of CHD*.^7^ Long-term consequences include prevalent behavioral problems and the need for remedial academic services.^8^ However, a meta-analysis of major surgical intervention trials to improve neurodevelopmental outcomes found that none were successful. Therefore, actionable perioperative factors are lacking to improve neurodevelopmental outcomes in neonates with CHD.^9,10^

Industrial chemical exposure in the health care environment is increasingly recognized as potentially harmful.^11,12^ Cyclohexanone is a major industrial solvent used in the production of adhesives, paint, plastics, and nylon.^13^ In animal toxic effects studies, inhalation of cyclohexanone resulted in central nervous system depression, as well as liver and kidney degeneration.^14,15^ This led to the establishment of National Institute for Occupational Safety and Health human inhalational exposure limits in the workplace. In health care, cyclohexanone is used as a bonding agent for joining medical plastic parts, such as intravenous (IV) tubing to stopcocks and IV bags.^16,17^ Cyclohexanone can easily migrate from polyvinyl chloride tubing and connections into standard IV fluids, such as saline, and cyclohexanone metabolites have been detected in urine samples from neonates receiving IV fluids.^18,19,20,21,22,23^ To our knowledge, the detection of circulating cyclohexanone in human cardiac surgical treatment and the potential pathophysiologic effects are unknown and could have a significant clinical impact. Accordingly, the objectives of this study were to determine the perioperative serum levels of cyclohexanone and its major metabolites in neonates undergoing cardiac surgical treatment with cardiopulmonary bypass (CPB) and to determine associations with neurodevelopmental outcomes at age 12 months.

## Methods

### Participants

This study is an ad hoc secondary analysis of the National Heart, Lung, and Blood Institute–funded Corticosteroid Therapy in Neonates Undergoing Cardiopulmonary Bypass randomized controlled trial of intraoperative methylprednisolone or placebo. The primary trial design and results have been reported following Consolidated Standards of Reporting Trials (CONSORT) reporting guideline (Primary Trial Protocol in Supplement 1).^24^ In brief, neonates younger than 1 month undergoing cardiac surgery with CPB and with 37 weeks postgestational age at the time of surgical treatment were enrolled at 2 participating centers between June 1, 2012 and November 21, 2017. Race/ethnicity data were assigned by the participant’s legal guardian. Participants were randomly assigned to either methylprednisolone at 30 mg/kg of body weight or placebo at the induction of anesthesia. At age 12 months, participants underwent neurodevelopmental testing. The protocol was approved by the institutional review board at the Medical University of South Carolina, and written informed consent was obtained from a parent or guardian before randomization (Primary Trial Protocol in Supplement 1). For this study, only enrollees between June 1, 2012, and October 31, 2016, at the Medical University of South Carolina who had completed neurodevelopmental testing at age 12 months were included. This secondary analysis was approved by the Johns Hopkins Institutional Review Board. This study is reported following the Strengthening the Reporting of Observational Studies in Epidemiology (STROBE) reporting guideline.

### Measurement of Neurodevelopment

The primary outcome was neurodevelopment assessment using the Bayley Scales of Infant and Toddler Development, third edition (BSID-III). The BSID-III is a validated screening tool used to assess neurodevelopment in children aged 1 to 42 months. The BSID-III was administered in-person by a single neuropsychologist who was blinded to treatment assignment and cyclohexanone levels during a follow-up study visit at age 12 months. The BSID-III assesses cognitive, language, and motor function with composite scores for each domain standardized to a population mean (SD) of 100 (15).

### Cyclohexanone and Metabolite Assays

Blood samples were collected from neonates at 3 points: preoperatively (prior to skin incision), postoperatively (immediately after CPB), and 12 hours postoperatively. Blood samples were held on ice until the serum was isolated, then aliquoted, and stored at −80 °C until assayed. To determine serum cyclohexanone and its metabolite concentrations, samples were spiked with isotopically labeled standards (Sigma-Aldrich) prior to acetonitrile extraction. All samples were analyzed within 48 hours of processing. Analysis of the samples was conducted on a 7010 GC-triple quadrupole mass spectrometer (Agilent) operating in electron-impact ionization mode (70eV source energy, 225 °C) and fitted with a Stabilwax analytical column (30 m × 250-μm diameter × 0.25-μm film thickness, 5-m integrated guard column, Restek #10623-124). Ultra high purity helium was used as a carrier gas, and ultra high purity nitrogen was used as a collision gas. Multiple reaction monitoring transitions for each target compound had been previously determined empirically by analysis of authentic standards. Peak integration and reporting was conducted using MassHunter Quantitative Analysis software version B.08.00 (Agilent). Retention times for all peaks were within 4 seconds of the expected values obtained from authentic standards. Quantitation was performed via an area ratio of cyclohexanone or its breakdown products to the internal standard area. All target compounds demonstrated a linear response across the concentration range of 1 pg to 1 ng on-column based on a dilution series of authentic standards. The percent coefficient of variability from the assay of a pooled plasma sample including 15 samples was cyclohexanone, 1.8%; cis 1,2 cyclohexanediol, 7.9%; trans 1,2 cyclohexanediol, 10.7%; 1,3 cyclohexanediol, 13.5%; and trans 1,4 cyclohexanediol, 15.2%. Samples that showed poor peak shape for the internal standard (apex >6 seconds from expected value) or showed chromatographic abnormalities were excluded from further analysis.

### Statistical Analysis

Standard descriptive statistics were used to summarize demographic, surgical, and clinical data. Continuous characteristics were listed as medians and interquartile ranges (IQRs). Categorical characteristics were expressed as number and percentage of participants. To determine if cyclohexanone levels at each time were associated with clinical demographic characteristics, Spearman correlation analysis (continuous variables), Mann-Whitney *U* tests (binary variables), or Kruskal-Wallis tests (categorical variables) were performed.

Values below the lower limit of detection (LLOD = 12 μg/L) for cis 1,2 cyclohexanediol (24%), trans 1,2 cyclohexanediol (1%), and trans 1,4 cyclohexanediol (1%) were replaced with the LLOD divided by √2.^25^ For each measurement point, the molar sum of the 4 cyclohexanone metabolites was calculated by adding their concentrations, each inversely weighted by its molecular weight, and multiplying by the molecular weight of cyclohexanone to express the sum in the same units as the parent compound.^26^ The geometric mean of the 3 measurements was calculated for cyclohexanone and the molar sum of its metabolites. Finally, each exposure variable was divided by its IQR to scale regression coefficients to a comparable difference in exposure level (ie, the 25th vs the 75th percentile).

For each composite neurodevelopment score (ie, cognitive, language, and motor), linear regression models were used to estimate unadjusted differences and 95% CIs per IQR increase in concentrations of the geometric mean of cyclohexanone or summed metabolites as well as concentrations at each time. Multivariable linear regression models were used to estimate differences adjusted for the clinically relevant clinical variables age and weight at surgical treatment, CPB time, Society of Thoracic Surgeons European Association for Cardio-Thoracic Surgery (STAT) mortality risk category (dichotomized as 1-3 vs 4-5), sex, age at neurodevelopment assessment, and steroid treatment. Regression diagnostics were examined to assess variance inflation and influential observations. Statistical analyses were performed with SAS version 9.4 (SAS Institute). *P* values were 2-sided, and associations were considered statistically significant at α = .05.

## Results

### Participant Demographic Characteristics

The flow diagram for the parent trial is shown in the Figure. Participant characteristics in this secondary study are described in Table 1. All 85 participants were at least 37 weeks gestational age at birth and underwent a cardiac operation with CPB. Approximately half of participants (40 participants [47%]) were randomized to methylprednisolone treatment. Most participants were boys (49 participants [58%]) and white (50 participants [59%]) or black (25 participants [29%]). As expected for neonatal cardiac surgical procedures, most participants had a STAT mortality risk category of 4 (48 participants [56%]) or 5 (17 participants [20%]). The median (IQR) CPB time was 194 (146-237) minutes, with deep hypothermic circulatory arrest used in a minority of patients (22 participants [26%]) and a median (IQR) arrest time of 8 (4-23) minutes. All participants received modified ultrafiltration. The mean (SD) age at neurodevelopmental assessment was 12.6 (0.7) months.

**Figure.**
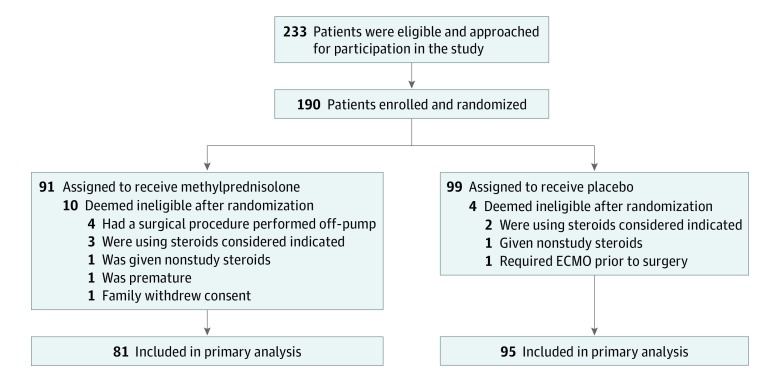
Parent Study Flow Chart ECMO indicates extracorporeal membrane oxygenation.

**Table 1.** Demographic, Surgical, and Clinical Characteristics of Included Participants

Characteristic	No. (%) (N = 85)
Gestational age at birth, median (IQR), wk	39.0 (38.4-39.8)
Boys	49 (58)
Race/ethnicity	
White	50 (59)
Black	25 (29)
Asian	2 (2)
American Indian or Alaska Native	1 (1)
Hispanic	6 (7)
Other	7 (8)
Procedure and diagnosis	
Corrective	54 (64)
Transposition of the great arteries	25 (29)
Aortic arch hypoplasia with ventricular septal defect	14 (16)
Truncus arteriosus	4 (5)
Other	12 (14)
Palliative	31 (36)
Hypoplastic left heart syndrome	12 (14)
Other single ventricle lesions	10 (9)
Tetralogy of fallot with pulmonary atresia	5 (6)
Other	3 (4)
Intraoperative characteristics	
Age at operation, median (IQR), d	8.0 (6-12)
Weight at operation, median (IQR), kg	3.3 (2.9-3.6)
STAT mortality risk category	
1	0
2	2 (2)
3	18 (21)
4	48 (56)
5	17 (20)
Cardiopulmonary bypass duration, median (IQR), min	194 (146-237)
Use of deep hypothermic circulatory arrest	22 (26)
Deep hypothermic circulatory arrest duration, median (IQR), min	8 (4-23)
Modified ultrafiltration	85 (100)
Clinical characteristics	
Methylprednisone treatment	40 (47)
Mechanical ventilation, median (IQR), d	4 (2.5-6.8)
Intensive care unit stay, median (IQR), d	8 (6.5-14)
Hospital stay, median (IQR), d	18 (11.5-33.8)

Abbreviations: IQR, interquartile range; STAT, Society of Thoracic Surgeons-European Association for Cardio-Thoracic Surgery.

### Cyclohexanone Serum Concentrations

A total of 6 preoperative samples and 6 samples from 12 hours after the operation that showed poor peak shape for the internal standard or showed chromatographic anomalies were excluded from further analysis. Thus, in samples that could be quantified, serum cyclohexanone levels were above the LLOD and quantified for 79 participants’ preoperation samples, all 85 participants’ immediately postoperation samples, and 79 participants’ 12 hours postoperation samples. Median (IQR) cyclohexanone levels were high in preoperation samples (572 [389-974] μg/L) point, increased more than 3-fold in the immediately postoperation samples (1744 [1469-2291] μg/L), and decreased, but were still quantifiable, in the 12 hours postoperation samples (146 [77-278] μg/L) (Table 2; eFigure 2 in Supplement 2).

**Table 2.** Serum Cyclohexanone and Cyclohexanone Metabolite Concentrations

Measure	Median (IQR), μg/L		
	Preoperative	Postoperative	12 h postoperative
Cyclohexanone	572 (389-974)	1744 (1469-2291)	146 (77-278)
Cyclohexanediol			
Cis 1,2	55 (0-113)	70 (15-130)	130 (0-208)
Trans 1,2	2155 (1413-3265)	1670 (1130-2680)	4139 (3223-5665)
1,3	490 (280-980)	520 (295-930)	1235 (760-1885)
Trans 1,4	1350 (690-1960)	970 (595-1400)	2265 (1585-3148)

Abbreviation: IQR, interquartile range.

### Cyclohexanone Metabolite Serum Concentrations

In samples that could be quantified, serum cyclohexanone metabolite levels were above the LLOD and quantified in 79 participants’ preoperation samples, all 85 participants’ immediately postoperation samples, and 79 participants’ 12 hours postoperation samples. Cyclohexanone was metabolized into the major breakdown products cis and trans 1,2 cyclohexanediol; 1,3 cyclohexanediol; and trans 1,4 cyclohexanediol. The highest median (IQR) concentrations of metabolites were found for trans 1,2 cyclohexanediol (preoperation sample: 2155 (1413-3265] μg/L; postoperation sample: 1670 [1130-2680] μg/L; 12 hours postoperation sample: 4139 [3223-5665] μg/L) and 1,4 cyclohexanediol (preoperation sample: 1350 [690-1960] μg/L; postoperation sample: 970 [595-1400] μg/L; 12 hours postoperation sample: 2265 [1585-3148] μg/L) and 1,3 cyclohexanediol (preoperation sample: 490 [280-980] μg/L; postoperation sample: 520 [295-930] μg/L; 12 hours postoperation sample: 1235 [760-1885] μg/L) (Table 2; eFigure 1 in Supplement 2). Unlike cyclohexanone, all metabolites peaked in the 12 hours postoperation sample, and the concentrations of trans 1,2 cyclohexanediol and trans 1,4 cyclohexanediol exceeded the concentration of the parent cyclohexanone compound at the preoperation and 12 hours postoperation samples. Concentrations of trans 1,2 cyclohexanediol and trans 1,4 cyclohexanediol decreased in the postoperation samples from preoperative levels but were increased in the 12 hours postoperation samples. There was a significant correlation of cyclohexanone levels in preoperation and postoperation samples (*r* = 0.435; *P* < .001) and in the immediately postoperation and 12 hours postoperation samples (*r* = 0.372; *P* < .001). There was a significant correlation of the Molar sum of the metabolites in the preoperation and postoperation samples (*r* = 0.356; *P* < .001) and in the immediately postoperation and 12 hours postoperation samples (*r* = 0.458; *P* < .001).

### Association of Cyclohexanone Serum Concentration With Clinical Variables

We found that higher cyclohexanone serum concentrations were significantly correlated with participants who had lower weight at surgical treatment (postoperation sample: *r* = −0.488; *P* < .001), lower gestational age at surgical treatment (postoperation sample: *r* = −0.315; *P* = .003; 12 hours postoperation sample: *r* = −0.266; *P* = .01), and younger age at surgical treatment (preoperation sample: *r* = −0.337; *P* = .001; postoperation sample: *r* = −0.276; *P* = .01; 12 hours postoperation sample: *r* = −0.367; *P* = .001) (Table 3). However, cyclohexanone concentrations were not associated with the STAT mortality risk category, CPB or deep hypothermic circulatory arrest duration, sex, or steroid treatment.

**Table 3.** Cyclohexanone Association With Clinical Demographic Characteristics

Variable^a^	Preoperative (n = 85)		Postoperative (n = 85)		12 h postoperative (n = 83)	
	*r*	*P* value	*r*	*P* value	*r*	*P* value
Weight at operation	−0.188	.09	−0.488	<.001	−0.139	.21
Gestational age						
At birth	0.012	.91	−0.148	.18	−0.036	.74
At operation	−0.187	.09	−0.315	.003	−0.266	.02
Age at operation	−0.337	.002	−0.276	.01	−0.367	.001
Duration, min						
CPB	0.142	.19	0.09	.40	0.165	.13
DHCA	0.236	.29	0.03	.89	−0.153	.50
STAT mortality risk category	NA	.63	NA	.28	NA	.06
Sex	NA	.71	NA	.90	NA	.45
Steroid treatment	NA	.67	NA	.24	NA	.96

Abbreviations: CPB, cardiopulmonary bypass; DHCA, deep hypothermic circulatory arrest; NA, not applicable; STAT, Society of Thoracic Surgeons-European Association for Cardio-Thoracic Surgery.

^a^ Continuous variables were analyzed with Spearman. Categorical variables were analyzed using Mann-Whitney *U* test, except STAT mortality risk category, which was analyzed with Kruskal-Wallis.

### Adjusted Associations of Cyclohexanone and Cyclohexanone Metabolite Levels With Neurodevelopmental Outcomes

In unadjusted analysis, cyclohexanone levels at the 12 hours postoperative point were significantly associated with lower cognitive, language, and motor composite scores (eTable in Supplement 2). In adjusted analysis, higher cyclohexanone levels at 12 hours after operation were associated with lower BSID-III composite scores for cognitive (β = −2.56; 95% CI, −4.23 to −0.88; *P* = .004), language (β = −2.27; 95% CI, −3.73 to −0.81; *P* = .003), and motor (β = −3.13; 95% CI, −5.35 to −0.90; *P* = .007) functions (Table 4). In addition, higher cyclohexanone geometric mean levels were associated with lower BSID-III composite scores for cognitive (β = −4.23; 95% CI, −7.39 to −1.06; *P* = .01) and language (β = −3.65; 95% CI, −6.41 to −0.88; *P* = .01) functions. The difference in composite scores for motor function among infants with higher cyclohexanone geometric mean levels was not statistically significant (β = −3.93; 95% CI, −8.19 to 0.33; *P* = .07). The 12 hour postoperation cyclohexanone metabolite molar sum was associated with a higher BSID-III composite score for language (β = 3.21; 95% CI, 0.45 to 5.98; *P* = .02) function.

**Table 4.** Adjusted Differences in Neurodevelopmental Composite Scores per Interquartile Range Increase in Concentrations of Cyclohexanone and the Molar Sum of Its Metabolites^a^

Measure	No.	Cognitive		Language		Motor	
		β (95% CI)	*P* value	β (95% CI)	*P* value	β (95% CI)	*P* value
Preoperation							
Cyclohexanone	85	−2.32 (−5.85 to 1.21)	.20	−1.14 (−4.25 to 1.97)	.48	1.71 (−2.95 to 6.38)	.47
Molar sum metabolites	82	0.07 (−3.04 to 3.17)	.97	−0.04 (−2.71 to 2.62)	.98	−0.82 (−4.83 to 3.19)	.69
Postoperation							
Cyclohexanone	85	1.01 (−2.68 to 4.70)	.59	0.98 (−2.25 to 4.21)	.55	−0.27 (−5.12 to 4.58)	.91
Molar sum metabolites	85	2.62 (−0.21 to 5.45)	.07	2.17 (−0.31 to 4.64)	.09	0.87 (−2.91 to 4.65)	.65
12 h postoperation							
Cyclohexanone	83	−2.56 (−4.23 to −0.88)	.004	−2.27 (−3.73 to −0.81)	.003	−3.13 (−5.35 to −0.90)	.007
Molar sum metabolites	80	2.90 (−0.28 to 6.09)	.08	3.21 (0.45 to 5.98)	.03	1.56 (−2.74 to 5.87)	.48
Geometric mean							
Cyclohexanone	83	−4.23 (−7.39 to −1.06)	.01	−3.65 (−6.41 to −0.88)	.01	−3.93 (−8.19 to 0.33)	.07
Molar sum metabolites	77	3.61 (−0.23 to 7.44)	.07	3.06 (−0.25 to 6.36)	.08	0.83 (−4.29 to 5.96)	.75

^a^ Adjusted for weight and age at operation, cardiopulmonary bypass time, Society of Thoracic Surgeons-European Association for Cardio-Thoracic Surgery category, sex, age at neurodevelopment assessment, and steroid treatment.

## Discussion

This secondary analysis of a randomized clinical trial found that neonates undergoing cardiac surgical treatment with CPB had high levels of serum cyclohexanone and its metabolites present prior to surgical treatment and had substantial increases after CPB. Importantly, higher serum cyclohexanone levels were independently associated with lower neurodevelopmental outcomes at age 12 months. To our knowledge, this is the first study to describe circulating cyclohexanone and metabolite concentrations associated with perioperative cardiac surgical exposure and suggest that IV cyclohexanone exposure has important health implications.

Cyclohexanone is an industrial solvent used as a bonding agent for joining medical plastic parts, such as IV tubing, to stopcocks and IV bags.^16,17^ Because of cyclohexanone’s heavy industrial use, there are decades of animal and some human toxicological data.^13^ Although cyclohexanone has been shown to leach from IV tubing, infusion sets, and importantly, CPB circuits, circulating cyclohexanone levels, its metabolism, and associated clinical outcomes are understudied.^18,19,20,21,22,23,24^ Because of its length, multiple stopcocks, and tubing connects, the CPB circuit in particular could be a significant reservoir for residual cyclohexanone. In a 2009 study,^23^ cyclohexanone was demonstrated to leach from CPB circuits with saline at levels of 210 to 698 μg/L. In our study, similar serum cyclohexanone levels were found preoperatively, likely from IV fluids. Following CPB, serum cyclohexanone concentrations increased 3-fold. In rats, a single IV bolus of cyclohexanone resulting in a serum concentration of 51 μg/L produced significant cardiovascular effects, including edema, systemic hypotension, pulmonary hypertension, and depressed cardiac contractility and output.^23^ We observed substantially increased preoperative and postoperative cyclohexanone concentrations in serum, likely associated with IV fluid and CPB circuits, in excess of what has been reported to have cardiovascular effects in animals.^23^

The National Heart, Lung, and Blood Institute has identified diminishing neonatal post-CPB brain injury as the most persistent and significant challenge to the current era of CHD care.^27,28,29,30^ However, to date, few actionable and generalizable perioperative factors have been identified with potential to reduce injury and improve outcomes.^31,32^ Our study found that higher serum cyclohexanone levels were independently associated with lower BSID III composite scores for cognitive, language, and motor function at age 12 months, suggesting that cyclohexanone exposure could be a contributor to neonatal cardiac operation–related neurodevelopmental outcomes. The neurotoxic effects of cyclohexanone have been well-documented in animal models, leading to decreased neural cell viability,^33,34^ neurological abnormalities,^35^ and death or moribund state.^34,35^ However, these studies used mature animals with variable concentrations of cyclohexanone and routes of administration, making direct comparison to perioperative IV cyclohexanone exposure challenging. Although there is evidence that cyclohexanone is toxic to the central nervous system, evidence for a minimum acute IV cyclohexanone dose, effects of cumulative exposure on neurodevelopmental outcomes, age-specific effects of exposure, or the mechanisms involved are unknown.

Cyclohexanone is at least partially metabolized by the liver and excreted by the kidney.^22^ Hepatic metabolism of cyclohexanone results in conversion to cyclohexanediol isomers (ie, 1,3; and 1,2; and 1,4 cis and trans).^36^ These alcohols are the major breakdown products of cyclohexanone and have been detected in urine from neonates exposed to standard IV bags and tubing.^22^ We found that cyclohexanone serum concentrations were inversely correlated with gestational age in our cohort. The decline in circulating cyclohexanone levels in the 12 hours postoperation samples, when IV fluids were still being administered, is likely associated with changes in cyclohexanone metabolism. We found that as circulating cyclohexanone levels declined, cyclohexanediols accumulated postoperatively and exceeded the concentration of the parent cyclohexanone at each time point. The accumulation of the cyclohexanediols could also be due to the long elimination half-times of cyclohexanediols (ie, 16-18 hours) demonstrated in urine after a single cyclohexanone inhalational exposure.^36^ In addition, daily repeated exposure has been shown to result in cumulative excretion, with similarity to increasing circulating cyclohexanediols at the 12-hour point in this study.^36^ The findings of our study suggest that metabolism of cyclohexanone to its major metabolites may be protective, as cyclohexanone metabolite levels were independently associated with better language composite scores. As the safety of the cyclohexanediols is still unclear, their benefit must be interpreted cautiously.

We found that neurodevelopment was not adversely associated with the cyclohexanone peak but with concentrations in the 12 hours postoperation sample. When we examined the geometric mean of cyclohexanone concentrations at all 3 time points, associations with worse neurodevelopment outcomes persisted, even with the adjusted analysis. Presently, it is unknown if cyclohexanone effects on the brain are due to aggregate exposure from receipt of IV fluids over the course of several days or exacerbated by prolonged exposure and then the large acute bolus from the CPB circuit. Expanded studies with more preoperative and postoperative measurements to clarify the actual perioperative peak and aggregate exposure are necessary to fully characterize and minimize cyclohexanone risk.

### Limitations

Our study has some limitations. Given financial constraints, this study used a subcohort of the parent trial with participants from a single center, which precluded our ability to determine the potential effect of center and vendor CPB circuit differences on cyclohexanone concentrations. By the nature of the parent trial’s inclusion and exclusion criteria, we could not determine whether the associations of cyclohexanone with outcomes would differ in younger neonates (ie, gestational age <37 weeks) or older infants and children. Additionally, we did not measure or control for exposure to other industrial contaminants, such as phthalates. Phthalate exposures increase after neonatal cardiac surgical treatment,^37^ and gestational exposures in the general population have been associated with neurodevelopmental outcomes in children.^38,39,40,41,42^

## Conclusions

The findings of this secondary analysis of a randomized clinical trail suggest that hospitalized neonates have significant circulating cyclohexanone exposure prior to cardiac surgical treatment and that circulating cyclohexanone levels increase dramatically after CPB. Cyclohexanone levels were independently associated with important neurodevelopmental outcomes in neonates, and gestational age may play an important role in its metabolism. Cyclohexanone exposure may have important health implications in infants undergoing surgical treatment for CHD and potentially anyone exposed to medical plastics. Cyclohexanone and plasticizers, such as phthalates, are likely ubiquitous in health care in the US, with exposure to IV fluids and medical devices, and could account for substantial national morbidity.

## Notes

Supplement 1. **Primary Trial Protocol**

Supplement 2. **eFigure 1.** Concentrations of Cyclohexanone and Its Metabolites in Serum **eTable.** Unadjusted Differences in Neurodevelopmental Composite Scores per Interquartile Range Increase in Concentrations of Cyclohexanone and the Molar Sum of Its Metabolites **eFigure 2.** Perioperative Cyclohexanone Concentrations

Supplement 3. **Data Sharing Statement**
