# Supplementary material for: Association of Neurodevelopmental Outcomes With Environmental Exposure to Cyclohexanone During Neonatal Congenital Cardiac Operations: A Secondary Analysis of a Randomized Clinical Trial
Source: JAMA Netw Open. 2020 May 6;3(5):e204070. doi: 10.1001/jamanetworkopen.2020.4070 (PMC7203603; doi:10.1001/jamanetworkopen.2020.4070)
Supplement: Supplement 1. — Primary Trial Protocol [file jamanetwopen-3-e204070-s001.pdf]

Supplemental Material

Pro # 16545

Corticosteroid Therapy in Neonates Undergoing Cardiopulmonary Bypass

(Steroids in Neonatal Cardiac Surgery)

Version 7.0 / Date: 09/15/2014 Grant# R01 HL112968

PRINCIPAL INVESTIGATOR

PRINCIPAL INVESTIGATOR
ERIC GRAHAM, MD
Associate Professor
MUSC
165 Ashley Avenue
MSC 915
Charleston, SC 29425
Phone:843-792-9570
Fax: 843-792-1978
E-mail:grahamem@musc.edu

BIOSTATISTICIAN

BIOSTATISTICIAN
RENEE' HEBERT MARTIN
Assistant Professor
MUSC
135 Cannon St.
Suite 303
Charleston, SC 29425
Phone:843-876-1913
Fax: 843-876-1923
E-mail:hebertrl@musc.edu

NHLBI PROGRAM OFFICER

ELLEN ROSENBERG
NHLBI
301-594-1376
rosenbeel@nhlbi.nih.gov

Protocol Synopsis

**Title**	Corticosteroid Therapy in Neonates Undergoing Cardiopulmonary Bypass
**Short Title**	Steroids in Neonatal Cardiac Surgery
**Clinical Phase**	Phase IV
**IND Number**	Exempt
**Rational for Study Population**	The study will focus on neonates for a few reasons. Although their post- cardiopulmonary bypass (CPB) clinical course is typically more severe and ICU care more prolonged than older children, their modes of morbidity are also well characterized. Further, the high level of severity itself provides a substrate for identifying the positive effects of a particular therapy. Finally, a therapy identified as beneficial has the greatest potential for benefit in this vulnerable population.
**Primary Aim**	Compare the effects of intraoperative methylprednisolone (MP) to placebo on morbidity and mortality following neonatal cardiopulmonary bypass (CPB).
**Hypothesis**	Neonates receiving MP will have an improved perioperative clinical course as assessed by a decrease in morbidity and mortality and evidence of capillary leak.
**Study Design**	Multi-center, prospective, randomized, double blind, placebo-controlled study of intraoperative MP in neonates undergoing cardiac bypass (CPB).<br><br>Randomization into one of the 2 study groups in the ratio of 1:1. The randomization will be stratified by corrective vs. palliative surgery and by surgeon.
**Primary Endpoint**	Incidence of a clinically derived composite morbidity-mortality outcome
**Secondary Endpoint**	- Inotropic score over the first 36 hours after surgery<br>- Incidence of low cardiac output syndrome over 36 hours<br>- Urine output over 36 hours<br>- Total intake/output of fluid over 36 hours<br>- Duration of mechanical ventilation, ICU and hospital length of stay<br>- Days alive & out of hospital at 90 days<br>- Bayley Scale of Infant Development at 1 year<br>- Inflammatory markers

	- Adverse Events
**Accrual Objective**	Two sites; 190 patients (95 patients per treatment arm)
**Study Duration**	5 year study
**Treatment Description**	Methylprednisolone (MP) at a dose of 30 mg/kg body weight will be administered intravenously with the induction of anesthesia
**Inclusion Criteria**	- Age ≤ 1 month (≤ 31 days of life)<br>- Male and female patients who are scheduled to undergo cardiac surgery involving CPB<br>- Parent or legal guardian consent
**Exclusion Criteria**	- Prematurity: < 37 weeks post gestational age at time of surgery<br>- Treatment with intravenous steroids within the two days prior to scheduled surgery.<br>- Participation in research studies involving the evaluation of investigational drugs or vaccines within 30 days of randomization.<br>- Suspected infection that would contraindicate steroid use (e.g. – Herpes)<br>- Known hypersensitivity to MP or one of its components or other contraindication to steroid therapy (e.g. - gastrointestinal bleeding).<br>- Preoperative use of mechanical circulatory support or active resuscitation at the time of proposed randomization.<br>- Inability to comply with study procedures

Table of Contents

Glossary of Abbreviations 7
1 BACKGROUND AND RATIONALE 8
1.1 Background 8
1.1.1 Glucocorticoid Prophylaxis for Post-CPB Inflammation 9
1.1.2 Methylprednisolone 9
1.1.3 Surrogate Measures of Low Cardiac Output 10
1.2 Hypothesis/Research Question 11
1.3 Preclinical and Clinical Experience 11
1.3.1 Clinical Experience 11
1.3.1.1 Inflammation after CPB 13
1.3.1.2 Cytokine Activation and Neonatal Post-CPB Inflammation 14
1.3.1.3 The cytokine response of neonates in general is known to b qualitatively and quantitatively distinctive in comparison to older infants. 14
1.3.1.4 Matrix Metalloproteinases (MMPs) and Neonatal Post-CPB Inflammation 15
1.3.2 Microribonucleic acid 16
1.3.3 Assessment of Inflammatory Markers 16
1.4 Rationale (for study population) 16
1.5 Known and Potential Risks and Benefits 17
1.5.1 Risk of Study Procedures 17
1.5.2 Benefits 18
2 AIMS 19
2.1 Primary Aim 19
2.2 Secondary Aims 19
3 SELECTION OF PARTICIPANTS 20
3.1 Inclusion Criteria 20
3.2 Exclusion Criteria 20
4 STUDY DESIGN 21
4.1 Study Endpoints 21
4.1.1 Primary Endpoint 21
4.1.2 Secondary Endpoint 21
5 STUDY TREATMENT REGIMEN 22
5.1 Premature Study Discontinuation 22
5.1.1 Premature Discontinuation of Study Treatment 22
6 CRITERIA FOR PREMATURE TERMINATION OF THE STUDY 23
6.1 Participant Withdrawal Criteria 23
7 STUDY PROCEDURES 24
7.1 Enrollment and Screening Procedures 24
7.1.1 Screening 24
7.1.2 Enrollment 24
7.1.3 Randomization 24
7.1.4 Intraoperative Management 25
7.1.5 Postoperative Management 25
7.1.6 Samples Collected 25
8 STUDY DRUG PREPARATION AND ADMINISTRATION 26
9 STUDY WINDOWS 27
10 SAFETY MONITORING 28
10.1 Definitions 28
10.1.1 Adverse Event 28
10.1.2 Serious Adverse Event 28
10.1.3 Study Deviation 29
10.2 Collection and Reporting of Events 29
10.2.1 Collection Procedure 29

10.2.2 Recording Procedure 29
10.2.3 SAE Grading and Attribution 29
10.2.4 Reporting Procedure and Timeline 30
10.2.5 Data Safety Monitoring Plan 30
10.2.6 Institutional Review Board Notification 31
11 RANDOMIZATION, BLINDING, UNBLINDING 32
11.1 Randomization 32
11.1.1 Randomization Code Development 32
11.1.2 Randomization Code Management 32
11.2 Blinding 32
11.3 Unblinding 33
12 STATISTICAL CONSIDERATIONS/ANALYTICAL PLAN 34
12.1 Sample Size and Power Calculation For Primary Endpoint 34
12.2 Sample Size and Power Calculation for Secondary Endpoints 34
12.3 Subgroup Analyses 34
12.4 Statistical Methods 35
12.5 Study Endpoint Assessment 35
12.5.1 Primary Endpoint 35
12.5.2 Secondary Endpoints 36
12.6 Study Data Collection 38
12.6.1 Table 2: Schedule of Measurements 38
12.6.2 Medical and Surgical History 40
12.6.3 Study Completion 40
12.7 Sample Size and Power Calculation For Primary Endpoint 40
12.8 Sample Size and Power Calculation for Secondary Endpoints 40
12.9 Subgroup Analyses 41
12.10 Statistical Methods 41
12.11 Reporting Deviations from Original Statistical Plan 42
13 SOURCE DATA 43
13.1 Identifying Source Data 43
13.2 Permitting Access to Source Data 43
14 QUALITY CONTROL AND QUALITY ASSURANCE 44
14.1 Data Monitoring Plan 44
15 ETHICAL CONSIDERATIONS AND COMPLIANCE WITH GOOD CLINICAL PRACTICE 45
15.1 Statement of Compliance 45
15.2 Informed Consent and Assent 45
15.3 Cost to Participate 45
15.4 Privacy and Confidentiality 45
16 PUBLICATION POLICY 46
17 REFERENCES 47

Appendix 1: Targeted /Planned Enrollment Table 53
Appendix 2: Schedule of Multiplexer Studies Table 54

Glossary of Abbreviations

CFR	Code of Federal Regulations
CRF	Case Report Form
CTCAE	Common Terminology Criteria for Adverse Events
DSMB	Data Safety Monitoring Board
FDA	Food and Drug Administration
GCP	Good Clinical Practice
ICH	International Conference on Harmonization
IND	Investigational New Drug
IRB	Institutional Review Board
NHLBI	National Heart, Lung, and Blood Institute
PI	Principal Investigator
SAE	Serious Adverse Event
SAP	Statistical Analysis Plan
ITT	Intent to treat
CPB	Cardiopulmonary Bypass
LCOS	Low cardiac output syndrome
ICU	Intensive Care Unit
MP	Methylprednisolone

1 BACKGROUND AND RATIONALE

1.1 Background

Cardiopulmonary bypass (CPB) has been characterized as "… a double-edged sword". Without it, cardiac surgery would not be possible in the majority of children with congenital heart disease. However, much of the perioperative morbidity that occurs after cardiac surgery can be attributed to pathophysiologic processes engendered by extracorporeal circulation" [1]. This observation is particularly true in the neonatal population, traditionally identified as infants less than 30 days of age. Although CPB per se is not an independent risk factor for mortality in neonatal heart surgery [2], the morbidity factors of smaller patient size, greater hemodilution, hypothermia, and longer CPB times combined with the biological immaturity of myocardial, renal, immune, hematological, gut and pulmonary systems create a post-bypass recovery period that is longer and more complex than similar operations performed in older infants and children [3, 4].

In all cases, CPB is a complex pathophysiologic environment in which exposure to non-physiologic surfaces in the pump circuit, hemolysis, and systemic and myocardial ischemia/reperfusion combine to create oxidative stress, reactive oxygen species (ROS) formation, peroxynitrite formation, cytokine release, complement cascade activation, matrix metalloproteinase (MMP) activation, and neutrophil activation[5, 6]. The result is a systemic inflammatory response (reviewed in [3, 4]). In the population of neonates undergoing CPB, the pathophysiology is complicated further by issues of size, but also more fundamentally by the biological immaturity factors noted above, a high likelihood of exposure to pre-operative prostaglandin E1 infusion, and coexistence of pre- and post-operative cyanosis and/or shunts. Cyanosis is known to increase the oxidative stress of CPB [7], and multiple studies have demonstrated qualitative and quantitative distinctions in cytokine release in children or neonates in response to CPB [4, 8-19].

Clinical appreciation of the post-CPB inflammatory response has resulted in a number of interventions directed at its reduction. Aprotinin administration, modification of pump circuit surfaces by heparin bonding, ultrafiltration strategies, leukocyte trapping filters, reduced oxygen exposure, monoclonal antibody administration, and glucocorticoid administration have all been described to reduce or eliminate clinical or biochemical features of the post-CPB inflammatory response in infants, children, or adults [7, 20, 21, 116]. Recent surveys by Checchia and Allen of national and international centers that perform pediatric CPB demonstrated that none of these interventions has achieved a level of standard practice in the pediatric or neonatal CPB population [21-23]. Of those responding to the surveys, glucocorticoid administration was the strategy applied by the largest number of centers; however, the timing, dosage and patient selection varied dramatically and reinforced the lack of convincing data to guide therapeutic administration of anti-inflammatory strategies for congenital heart surgery [21-23].

This application proposes a randomized controlled trial of the use of glucocorticoids to improve the clinical course of neonates post-CPB. The study will focus on neonates for a few reasons. Although their post-CPB clinical course is typically more severe and ICU care more prolonged than older children, their modes of morbidity are also well characterized. Further, the high level of severity itself provides a substrate for identifying the positive effects of a particular therapy [24].

Finally, a therapy identified as beneficial has the greatest potential for benefit in this vulnerable population. A composite morbidity-mortality outcome consisting of six individual clinically relevant criteria (death, cardiac arrest, ECMO, renal insufficiency, hepatic insufficiency, and lactic acidosis) will be used as the primary endpoint, while a variety of secondary endpoints will be related to the biochemical anti-inflammatory effects of therapy, ICU care and late neurological outcomes.

1.1.1 Glucocorticoid Prophylaxis for Post-CPB Inflammation

The theoretical rationale for the use of glucocorticoids in the setting of CPB is their ability to diminish production of inflammatory mediators by transcriptional mechanisms [25, 26]. This mechanism of action is not therefore directed at the initiating events in the inflammatory sequence (ischemia/reperfusion based generation of ROS, activation of complement/neutrophils/platelets in the CPB circuit, hemolysis), but on the reduction of the responsiveness of the immune system to these stimuli. Perioperative steroids have been demonstrated in animals and/or humans to improve oxygen delivery, increase pulmonary compliance, lower body temperature, reduce fluid requirements, and decrease the post bypass inflammatory response [27-29].

Few controlled trials of glucocorticoids in the setting of CPB in children can be found [22, 27-32]. Two of these studies involving only 29 patients each suggest some evidence for improvements in clinical parameters such as ICU stay and fluid requirement [27, 29]. However, other studies including a recent meta-analysis failed to demonstrate any benefit in clinical outcomes with glucocorticoid administration [32, 33]. The conclusions from all these trials are limited by inadequate sample size, inconsistent steroid agent or dose, and timing differences. It is important to note that glucocorticoids have a wide variety of deleterious effects, including diminished wound healing and anti-immune actions that make them undesirable in the absence of a clear clinical indication.

In the largest pediatric study, Pasquali et al. utilized the Pediatric Health Information Systems Database to evaluate the outcomes of corticosteroids in children 0-18 years of age undergoing congenital heart operations [34]. Data from 46,730 patients were included, 54% of which received corticosteroids. Multivariable analysis found no difference in postoperative mortality between corticosteroid recipients and non-recipients. Corticosteroids were associated with longer length of stay, greater infection, and greater use of insulin. In a sub-analysis of 10,018 neonates, corticosteroids were associated with significantly longer postoperative ICU length of stay and greater use of insulin. Although this study has the inherent limitations of any observational study utilizing a large database and the authors were unable to evaluate the impact of different dosing regimens or exact timing or corticosteroid administration in relation to surgery it highlights the importance of confirming these findings in an adequately powered randomized trial prior to any firm clinical recommendations.

1.1.2 Methylprednisolone

Methylprednisolone (MP) given intravenously is a potent, synthetic glucocorticoid that has powerful anti-inflammatory effects. MP is extremely water-soluble and may be administered in a small volume of diluent. This is particularly advantageous for neonates and small infants

whose cardiovascular system will not tolerate infusion of large volumes of intravenous fluid. The optimal type of steroid and steroid dose for use with CPB remains controversial; however, MP was selected at the dose of 30 mg/kg because it is approved for use in children, is the most commonly used glucocorticoid in the setting of cardiac surgery and has been used in other small pediatric studies without reported important side-effects [5, 21, 29, 35].

1.1.3 Surrogate Measures of Low Cardiac Output

The predictable and reproducible fall in cardiac output/index (CO/CI) that occurs after CPB for congenital heart surgery, particularly in neonates, infants and young children is well documented. In 1975, Parr and associates [36] reported that nearly 25% of young children after CPB had a CI of < 2.0 L/min/m^2 and concluded that low CI was a strong predictor of acute cardiac death. Similarly, in 1995, Wernovsky and colleagues[37] found that 25% of neonates had a CI of < 2.0 L/min/m^2, typically occurring between 6 and 18 hours after surgery. This predictable fall in CI was associated with an elevation of systemic vascular resistance of ~ 25% over baseline, and a rise in pulmonary vascular resistance of ~ 40% over baseline. Other recent reports have documented similar, predictable falls in CI and elevations in systemic and pulmonary vascular resistance following surgery in neonates[38, 39]. This low CI state has been referred to and subjectively defined as low cardiac output syndrome (LCOS) [24]. The cause of low cardiac output is multifactorial. Surgical repair of cardiac malformations with CPB exposes the myocardium to prolonged periods of myocardial ischemia[40] and cardioplegia[41]. Some repairs require a ventriculotomy, with resultant myocardial dysfunction. Finally, following surgery, there are acute changes in the loading conditions of the myocardium. Infants with cardiac malformations account for 80-90% of the pediatric patients who develop low cardiac output[42]. Some cases of low cardiac output after congenital heart surgery are due to residual hemodynamic burdens of the underlying defect; however, even when all anatomical defects are repaired there is still depressed myocardial performance that is associated with increased mortality[36]. While mortality from low cardiac output has declined considerably in recent years, it is still accompanied by increased morbidity, including: prolonged invasive monitoring, cardiac support, ventilatory support and ICU stay, seizures, long-term cognitive dysfunction and multiorgan dysfunction [22, 37, 43, 44].

The diagnosis of low cardiac output after cardiac surgery may be problematic. Objective measurements of CI may not be possible in patients with residual intracardiac shunts, and technical considerations generally limit the use of thermodilution techniques in small neonates and infants. Alternative objective assessments include the measurement of mixed venous oxygen saturations and serum lactate levels. Parr [36] showed a close correlation between low mixed venous oxygen saturations and low CI measured by thermodilution in young patients following cardiac surgery. Recent studies have confirmed that a rising arterial-venous difference in oxygen (A-V DO_2) is correlated with low CI and an increased likelihood of adverse hemodynamic events. Serum lactate has also been increasingly utilized to predict low cardiac output. Studies have shown that a rising lactate predicts mortality or the need for extracorporeal membrane oxygenation (ECMO) with 89% sensitivity, 100% specificity, and 100% positive predictive value in neonates after CPB [45].

Given the subjectivity of diagnosing low cardiac output and that death occurs rarely in our study population a composite morbidity-mortality outcome will be the Primary Endpoint. The

morbidity-mortality composite outcome consists of six objective individual criteria. Subjects will meet the outcome if they have one or more of the following after surgery and before hospital discharge: death, cardiac arrest, need for extracorporeal membrane oxygenation, renal insufficiency (creatinine more than two times normal), hepatic insufficiency (aspartate aminotransferase or alanine aminotransferase more than two times normal >36 hours post-op), or a rising lactic acidosis (>5mmol/L). [117] Similar morbidity-mortality composite outcomes have been used in other pediatric cardiac surgery studies[107,114,115].

Congenital heart disease is the most common cause of birth defects, with about 40,000 new cases born per year in the US. An estimated 10,000 of these patients will undergo cardiac surgery involving CPB. Furthermore, it is estimated that over 300,000 children in the US under age 21 have congenital cardiovascular disease and that 38% of these children will have had one or more surgical procedures (AHA). The use of CPB in neonates in particular has increased steadily over the past two decades. Further, neonates are generally sicker and consume more resources, including postoperative mechanical ventilation, ICU stay and hospital stay. Consequently, reducing the deleterious effects of CPB will have the largest impact in this group of patients. The "costs" of low cardiac output and associated morbidity after CPB involve all aspects of the child, family, and community. The prolonged ICU and hospital stays expose the infant to a variety of hospital related complications in addition to long term neurodevelopmental delay, while stressing the family emotionally and financially. Based on the frequency of complex congenital heart defects, the incidence of the low cardiac output, and the daily costs of ICU care, a reduction in postoperative morbidity can be estimated to decrease total healthcare expenditures in the US by hundreds of millions of dollars annually.

1.2 Hypothesis/Research Question

Neonates receiving MP will have an improved perioperative clinical course as assessed by a decrease in morbidity and mortality and evidence of capillary leak.

1.3 Preclinical and Clinical Experience

1.3.1 Clinical Experience

Given the rationale of glucocorticoid administration in the setting of CPB is targeted at the reduction of the responsiveness of the immune system to the CPB stimuli, the preventive effect of glucocorticoids would be presumed to be augmented by administration prior to the inflammatory stimulus. Animal research [46] and small clinical observations [29] support this presumption. However, recent work by Dr. Graham (PI) challenged this canonical belief and provides the rationale for this investigation [5]. Neonates scheduled for cardiac surgery were randomly assigned to receive either Two Dose (8 hours preoperatively and operatively; n=39) or Single Dose (operatively; n=37) methylprednisolone (MP; 30 mg/kg/dose) in a prospective, double-blind, controlled trial. The primary outcome was the incidence of low cardiac output syndrome or death 36 hours postoperatively. Secondary outcomes were death at 30 days, interlukin-6 levels, inotropic score, fluid balance, serum creatinine, and ICU and hospital stay. Preoperative plasma levels of the inflammatory cytokine interlukin-6 were reduced by 2-fold (p<0.001) in the Two Dose MP group, consistent with the anti-inflammatory effects of preoperative MP administration. However, the incidence of low cardiac output syndrome was 46% (17/37) in the Single Dose and 38% (15/39) in the Two Dose MP groups (p=0.51). Two

Dose MP was associated with a higher serum creatinine (0.6 ± 0.2 vs. 0.5 ± 0.1 mg/dL, p=0.03), and poorer postoperative diuresis (-96±49 mL, p=0.05). Inotropic requirement, duration of mechanical ventilation, ICU, and hospital stay did not differ between the 2 groups. In this, the largest pediatric and only exclusively neonatal randomized trial comparing the efficacy of preoperative glucocorticoid therapy to intraoperative glucocorticoid therapy alone for neonates undergoing cardiac operations requiring CPB, our data did not provide support for the addition of a preoperative dose of MP to a standard intraoperative dose alone. Although preoperative dosing was associated with an improvement in preoperative inflammatory markers, this did not correlate with improved clinical outcomes. To the contrary, preoperative MP dosing was associated with a higher immediate postoperative serum creatinine level, and worse diuresis and fluid balance over the first 36 hours postoperatively. In light of these findings, the routine use of preoperative glucocorticoids in neonatal cardiac surgery needs to be reconsidered.

However, the conclusions of this study were significantly limited by the lack of a true placebo group. Thus recommendations for or against intraoperative MP could not be made. At the time of study design, in light of the presumed beneficial effects demonstrated in both animal and small pediatric studies and given the widespread use of MP, we considered it inappropriate to randomize neonates to a completely placebo control group. The widespread use and our decision to exclude a completely placebo arm was further confirmed in the recently published trial comparing shunt types used in the Norwood surgery [47]. Of the 549 patients undergoing the surgery, 498 (91%) received some type of steroid during the surgery (unpublished data 2010, Pediatric Heart Network). The manuscript of this work received favorable reviews from the Journal of the American Medical Association but was ultimately rejected given the lack of a placebo group. Considered in total, the utility of intraoperative steroids remains in question. A randomized trial comparing intraoperative glucocorticoid to placebo is indicated.

Dr. Graham has investigated the commonly used outcomes of LCOS and vasoactive inotropic score with respect to the strength of associations to clinical outcomes following neonatal cardiac surgery[113]. The important findings were that LCOS, as defined in the PRIMACORP study, was not associated with early postoperative outcomes in neonatal cardiac surgery. However, maximum vasoactive inotropic score was correlated with the duration of mechanical ventilation (r=0.36, p=0.001) and ICU stay (r=0.27, p=0.02). This study questions the problematic and subjective definition of LCOS. Given this, the objective composite morbidity-mortality outcome will be the Primary Endpoint in this proposal. Similar composite morbidity-mortality outcomes have been used in other studies in children following cardiac surgery[107,114,115]. When this composite outcome was applied to the subjects in Dr. Graham's initial steroid trial, 33% (25/76) of the study population met the composite morbidity-mortality outcome. Subjects that met the composite outcome, when compared to those that did not, had significantly longer durations of mechanical ventilation, ICU and hospital stays (Table 1,). [117] This provides additional support to the clinical relevance of this objective composite outcome and superiority to LCOS as a surrogate marker for assessment of low cardiac output in this population.

Table 1. Composite Morbidity-Mortality Outcome and other Postoperative Outcomes

	Composite Outcome - No n=51	Composite Outcome - Yes n=25	p-value
Mechanical ventilation (days)	4.0 ± 3.9	11.1 ± 13.0	0.01
Intensive care unit stay (days)	7.0 ± 4.5	18.8 ± 25.1	0.03
Hospital stay (days)	16.4 ± 9.0	34.6 ± 29.8	0.01
Hospital charges ($10,000)	19.5 ± 6.9	35.2 ± 26.2	0.01

Data are reported as mean ± SD.

Given unclear benefits and potential detrimental effects, a randomized trial comparing intraoperative glucocorticoids to placebo is mandatory. Dr. Graham is uniquely qualified to lead this study. He was the principal investigator evaluating preoperative and intraoperative MP to intraoperative MP alone in neonates undergoing cardiac surgery, which provides the rationale for the current proposal [5]. Dr. Graham has proven collaboration with the Departments of Surgery, Anesthesiology and Perioperative Medicine and Biostatistics and Epidemiology to sucessfully complete this project. His work has also placed the Medical University of South Carolina in a unique position to perform this proposal. Despite conflicting data, the use of perioperative glucocorticoids remains prevelant [21, 23]. Dr. Graham's prior work has resulted in an institutional equipoise in regard to perioperative glucocorticoid administration, highlighted by the uniform support of this proposal by the surgical and ICU physicians.

Our efforts in this application are to specifically study glucocorticoids, but in the larger picture we are establishing a platform for a comprehensive understanding of CPB-mediated inflammation in the immature or cyanotic myocardium, in order to better suggest and evaluate future myocardial protective strategies. As an example, in a closely related project, our group (Drs. Graham, Atz and Bradley) have investigated matrix metalloproteonases (MMP) activity and cytokine signaling, and possible modification by antioxidant drugs, in older infants with tetralogy of Fallot or ventricular septal defects [6]. The hypothesis is that preoperative cyanosis will result in increased sensitivity to oxidative stress, resulting in higher MMP activity and increased pro-inflammatory cytokine levels. We have found that serial profiling a large array of cytokines and proteolytic enzymes after surgery for congenital heart disease can provide insight into relationships between changes in bioactive molecules to early postoperative outcomes and may hold significance as biomarkers for predicting and managing the postoperative course [5, 6, 48].

1.3.1.1 Inflammation after CPB

Mann [49] stated that "the inflammatory cascade is hierarchical, intertwined, and redundant". The cascade includes pro-inflammatory cytokines, anti-inflammatory cytokines, MMPs, and products of neutrophil activation. If glucocorticoid therapy benefits the neonatal population it is most likely to be through modification of the inflammatory cascade. This proposal seeks to compare the biochemical profile of the post-CPB state in neonates with respect to a panel of the best characterized pro-and anti-inflammatory cytokines as well as the completely uncharacterized microribonucleic acid (miRNAs) and matrix metalloproteinase response.

1.3.1.2 Cytokine Activation and Neonatal Post-CPB Inflammation

CPB results in myocardial ischemia/reperfusion. This in turn causes oxidative stress and the formation of reactive oxygen species (ROS). The immature myocardium seems more vulnerable to oxidative stress than adult myocardium, and the degree of pre-existing cyanosis may significantly contribute to the ROS mediated myocardial dysfunction [50-52]. Oxidative stress precedes the release of cytokines during congenital cardiac surgery, indicating that ROS may be important initiators of a biological cascade that can culminate in myocardial dysfunction and low cardiac output [50, 53, 54]. Hemolysis, presumably from the CPB circuit and measured by plasma free hemoglobin levels, was recently indicated to be an especially important marker in predicting post-CPB clinical course, perhaps through its potent oxidative effects [54].

In general terms, cytokines are produced by multiple cell types, including cardiac myocytes and fibroblasts, and bind to receptors on multiple cell types, activating transcription factors such as nuclear factor kappaB (NFkB) and AP-1 [55-58] Cytokines released in the context of ischemia/reperfusion and oxidative stress include tumor necrosis factor alpha (TNFα) and the interleukins (ILs) IL-2, IL-6, IL-8, and IL-10 [4, 7, 15-18, 30, 50, 51, 53, 54, 59-71]. TNFα has been clearly demonstrated to be released during cardiac surgery and CPB. Through binding to transmembrane receptors, it causes the activation of several transcription factors – notably NFkβ [57, 58]. IL-2, IL-6 and IL-8 are pro-inflammatory cytokines that mediate the activation and maturation of inflammatory cells and thereby the augment release of ROS and proteases beyond that initiated by the original ischemia-reperfusion insult [3, 62-64, 72]. IL-1β is also pro-inflammatory, potently recruiting inflammatory cells and inducing proteases [3, 73, 74]. IL-1β forms another potent pathway for NFkβ formation has been reported following congenital heart surgery [60, 61, 75, 76]. However, the overall impact of this potent cytokine to downstream effector systems remains to be established. Chemokines (eg - MCP-1) augment the egress and maturation of inflammatory cells, such as neutrophils, into the myocardium and thereby facilitate cytokine activation [77, 78]. In contrast to the pro-inflammatory ILs, anti-inflammatory cytokines, such as IL-10, appear to inhibit further cytokine release from multiple cell types [3, 79]. The balance of pro- and anti-inflammatory cytokines is believed to determine the degree of inflammatory response. It is clear that a wide array of molecules play a role in the post-CPB response, but equally clear that our understanding of these roles can only be derived from further clinical studies.

Modified ultrafiltration (MUF) is commonly employed at the conclusion of CPB in order to reduce the impact of hemodilution [17, 60, 71, 72, 75, 76, 80, 81]. MUF has been shown to transiently affect cytokine levels, but cytokine activation continues unabated in the early post-operative period [81]. Moreover, TNF and IL-8 levels appear to be relatively unaffected by MUF, whereas IL-10 levels are reduced - potentially contributing to a 'feed-forward' cytokine activation process in the early post-CPB period [4, 57, 60].

1.3.1.3 The cytokine response of neonates in general is known to b qualitatively and quantitatively distinctive in comparison to older infants.

A small number of studies have specifically examined the neonatal cytokine response to CPB as distinguished from older infants and pediatric patients [8, 16-18, 71]. In general, investigators have uniformly noted augmentation of IL-10 levels when measured and variability of IL-6, IL-8, and TNF alpha responses. Preoperative glucocorticoid administration in children[29] and

hypothermia on bypass[19] augment these profiles, while intraoperative PGE1 administration in adults on CPB result in similar cytokine profiles [82]. In summary, there is increased IL-10 with high variation in reported pro-inflammatory cytokine levels in neonates, and common practices in neonatal preoperative or intraoperative care may influence these balances.

1.3.1.4 Matrix Metalloproteinases (MMPs) and Neonatal Post-CPB Inflammation

MMPs are a family of at least 25 zinc-dependent proteins that were classically associated with their ability to degrade collagens or other extracellular matrix macromolecules. These properties are important features of MMP function, but intensive investigation has revealed much broader roles for these proteins in the myocardium and in other tissues. MMP degradation products of collagens and other macromolecules often have significant biological activities, and MMPs also mediate the activity of many biologically important growth factors (e.g., IGF family members, TGFβ family members [83, 84]). MMPs have become a very active arena of investigation in the adult heart. MMPs have been recognized to increase in the bronchoalveolar lavage fluid of pediatric patients, including neonates, with lung disease [85-87]. Serum MMP levels have been altered in premature infants with bronchopulmonary dysplasia or interventricular hemorrhage [85], and increased MMP-3 (stromelysin-1) was found in the non-necrotic gut of infants with necrotizing enterocolitis [88]. Altogether, however, few studies of MMP levels have been performed in the neonatal population or the immature heart.

Two reports have found elevation of MMP-2 in the heart after asphyxia-induced cardiac ischemia in piglets, with no changes in MMP-9 [52, 89]. There are currently no clinical data for neonates, infants, or older children to predict the nature of the MMP response post CPB. However, Cheung et al. studied neonates on ECMO and were able to show that plasma MMP-2 was elevated in a time-dependent fashion, but not MMP-9 [90], and the adult data suggest MMP changes are important in the response to a variety of cardiac stresses and injuries. Induction of MMPs has been found in patients following myocardial infarction, in ischemia/reperfusion, and most importantly in the context of cardiac surgery [77, 78, 91-99]. Importantly, a cause-effect relation between MMP induction and changes in myocardial structure and function has also been demonstrated in the context of ischemia/reperfusion [99-102]. However, a quantitative assessment of MMP profiles in congenital cardiac surgery and the inter-relationship to postoperative clinical status and cytokine activation has not been performed.

The present study will examine a cassette of MMP types corresponding to three important soluble MMP classes: the interstitial collagenases (MMP-1,-8,-13), gelatinases (MMP-2,-9) and matrilysin/stromelysin (MMP-3,) using a multiplexer approach which has been optimized, validated and calibrated for each of these MMP species. As published reports describe an apparent cell and stimulus-specific effect of PGE1 on production of MMPs [103], the net effect of PGE1 administration as a confounding variable on total circulating MMP levels in neonates pre- and post-CPB is difficult to predict. It is likely that the effects on augmentation or suppression of individual MMPs in this setting will be tissue specific. Our study will provide the initial clinical data for further investigation of this topic.

1.3.2 Microribonucleic acid

Microribonucleic acid (miRNAs) are a recently discovered small, non-coding RNAs that regulate protein levels post-transcriptionally[104]. miRNAs play important regulatory roles in many cellular processes, including differentiation, neoplastic transformation, apoptosis and cell replication and regeneration. Given these regulatory roles, it is not surprising that miRNA expression has been implicated in numerous disease states. Recent studies have reported significant levels of miRNAs in serum and other body fluids, raising the possibility that circulating miRNAs could serve as useful clinical biomarkers. Even more interesting, given their control over multiple effectors of complex intracellular pathways make miRNAs tremendously attractive as therapeutic targets, distinguishing them from single protein targets (i.e. enzyme or receptor) of most conventional drugs[105]. Various biochemical means of stabilizing and delivering miRNAs antagonists have already proven successful. miRNAs role in cardiovascular disease is still incompletely understood and under investigation[106].

1.3.3 Assessment of Inflammatory Markers

Until recently, studying markers for all of these processes has been a practical impossibility in neonates due to the volume of blood sampling needed and the specialized nature of the measurements. However, recent technical advances included in this application make it realistic to match clinical outcomes with the time course of a broad array of serum pro-and anti-inflammatory cytokines, MMPs, myeloperoxidase and miRNAs. These molecules, known as analytes can all be measured from the same small blood sample by a process called multiplexing. The multiplex analysis technology in this proposal was developed commercially by Luminex Corporation and combines the sensitivity of enzyme-linked immunosorbent assays with the specificity and multiplexing capability of flow cytometric detection. The equipment described in this proposal enables up to hundreds of different analytes to be measured within a single sample of less than 50 microliters of plasma. For the purposes of this proposal we will measure the more selected panel of MMPs, cytokines and miRNAs detailed in Appendix 2.

1.4 Rationale (for study population)

The study will focus on neonates for a few reasons. Although their post- CPB clinical course is typically more severe and ICU care more prolonged than older children, their modes of morbidity are also well characterized. Further, the high level of severity itself provides a substrate for identifying the positive effects of a particular therapy. Finally, a therapy identified as beneficial has the greatest potential for benefit in this vulnerable population.

The primary impediment to performing mechanistically based controlled studies in the area of congenital heart surgery is the complexity and diversity of the lesions and ages that confront the pediatric cardiologist and surgeon. However, the conditions that require neonatal cardiac surgical intervention are all relatively severe, and the responses of neonates to cardiopulmonary bypass (CPB) are relatively uniform and well defined. Further, the volume of neonates undergoing CPB is quite large, providing adequate sample size to test a focused intervention and set of hypotheses. The reductionist approach of this project in regards to the complexity of the congenital heart malformations and surgical procedures has been chosen to balance the objectives of scientific rigor, acceptable volume and clinically meaningful endpoints. It is recognized that a large number of patients are not being considered in this initial proposal, such

as infants and older children, where it is also likely that post bypass injury is an important problem. However, inclusion of only the neonatal subset of patients will decrease the complexity of the study design, allowing for a sharp and mechanistic focus.

1.5 Known and Potential Risks and Benefits

1.5.1 Risk of Study Procedures

The surgery and post-operative recovery to discharge will follow standard facility SOPs and be based upon the decision of the primary physician caring for the neonate.

Risks related to the study include:

- Risk of Methylprednisolone administration:

The possible risks or discomforts of the study are minimal. Intravenous steroids are widely used for a myriad of inflammatory conditions including cardiopulmonary bypass. When steroid treatments are given for repeated doses, they may cause important disturbances in the stomach or intestine (e.g. ulcers), pancreas, salts and sugar in the blood, or the ability to fight infection.

- Risk of Infection due to Sampling:

This risk is quite small. The risks associated with blood collection are minimal since an arterial and venous catheter will be in place for routine clinical use. Thus, any risks associated with a second venipuncture are eliminated. The volume of blood drawn for these studies has been carefully considered to minimize the potential risk of reducing circulatory volumes in the post-operative period. The risk associated with the intra-operative samples is minimal since volume supplementation occurs during this period as a matter of course for the conduct of cardiopulmonary bypass. The blood samples at later time points have been carefully considered and have been spread over a 30-hour period. It is standard of care to use a urine catheter and the risk of acquiring and infection resulting from urine collection is minimal.

- Risk of Developmental Testing

The evaluation performed by the developmental specialist at one year of age does not involve any invasive testing. This portion of the study will take approximately one hour of time. There are no risks associated with this evaluation.

- Risk of loss of confidentiality:

The risks with hemodynamic and postoperative data do not constitute health risks, but rather those of data security and confidentiality. Confidentiality will be maintained by making the study data available to only the investigators, and by omitting specific patient information (name, hospital number) from any published data.

- Protection against Risk:

All parent(s)/legally authorized representatives (LAR), (their families and their personal physicians where appropriate), will be informed in advance of executing the informed consent document of all potential risks attendant to participation in study protocol as described above. Throughout the performance of this study and during the performance of all procedures, subjects will be carefully monitored in accordance with current medical practices. Treatment with a single

dose of MP as proposed in this study has had few if any reported side effects. Patients whose parent(s)/legally authorized representatives (LAR) refuse consent or who are not enrolled in this study may receive intraoperative MP, based on their attending cardiothoracic surgeons or anesthesiologist preference. Based upon the extensive experience with all aspects of the study protocol procedures, it is anticipated that that the risk of adverse events associated with the study will be acceptably low. Maximum efforts will be undertaken to ensure the safety of all study participants. All personnel involved in performance of the study and its procedures will be fully trained and extensively experienced. All serious adverse events will be required to be reported to the local IRB per local protocol within 24 hours of it being brought to the attention of the attending physician of the patient as well as the principal investigator of the study. Other (non severe) adverse events will be reported according to local IRB protocol. In accordance with the current revision of the Declaration of Helsinki and other applicable regulations, a patient (or patient's parent(s)/legally authorized representatives (LAR)) has the right to withdraw the patient from the study at any time and for any reason without prejudice to patient's future medical care by the physician or at the institution. Should a patient (or patient's parent(s)/legally authorized representatives (LAR)) request or decide to withdraw from the study, all efforts will be made to complete and report the observations as thoroughly as possible through the date of withdrawal and the protocol-related observations for the last visit. If the patient is withdrawn due to an adverse event, the Investigator is to arrange for the patient to have follow-up visits until the adverse event has resolved or stabilized in addition to the evaluations required for the end of the study. Confidentiality will be maintained by making the study data available to only the investigators, and by omitting specific patient information (name, hospital number) from any published data. Appropriate HIPAA consents and procedures will also be employed to ensure confidentiality.

1.5.2 Benefits

There is no known, direct benefit to participation. Neurodevelopmental testing at 1 year of age is not standard of care at all institutions, but is a secondary endpoint in this study. Many families would consider this evaluation by a developmental specialist at a year of age a benefit.

2 AIMS

2.1 Primary Aim

- Compare the effects of intraoperative methylprednisolone (MP) to placebo on morbidity and mortality following neonatal cardiopulmonary bypass (CPB).

2.2 Secondary Aims

- Compare the effects of MP to placebo on the duration of mechanical ventilation, ICU and hospital stay post cardiac surgery.
  Hypothesis: Neonates receiving MP will have decreased morbidity and capillary leak which will result in shorter ICU and hospital stay.
  Endpoints:
  Time of mechanical ventilation, ICU stay, and total hospital stay
  Number of days alive and out of the hospital at 90 days post cardiac surgery

- Compare the effects of MP to placebo on neurologic and developmental outcomes at 1 year.
  Hypothesis: Neonates receiving MP will have improved neurologic and developmental outcomes at 1 year.
  Endpoints: Bayley Scale of Infant Development at 1 year

- Compare the effects of MP to placebo on the inflammatory response to cardiopulmonary bypass.
  Hypothesis: Neonates treated with MP will have altered production of inflammatory cytokines and other biomarkers of organ dysfunction.
  Endpoints:
  - Multiple agents serially measured preoperatively and following CPB by novel multiplexer technique including but not limited to:
  - Cytokines
  - Matrix metalloproteinases (MMPs)
  - Microribonucleic acid (miRNAs)
  - Endothelial function

- Compare the safety of MP to placebo following cardiac surgery.
  Hypothesis: Neonates treated with MP will have a similar rate of adverse events over the first 30 days after surgery.
  Endpoint:
  - Incidence of adverse events and serious adverse events
  - Rise in creatinine and incidence of acute kidney injury

3 Selection of Participants

3.1 Inclusion Criteria

- ≤ 1 month of age (≤31 days of life)
- Male and female patients
- Scheduled to undergo cardiac surgery involving CPB
- Consent obtained from parent(s)/Legally Authorized Representative (LAR)

3.2 Exclusion Criteria

Patients who meet any of these criteria are not eligible for enrollment as study participants

- Prematurity: < 37 weeks post gestational age at time of surgery
- Treatment with intravenous steroids within the two days prior to scheduled surgery.
- Participation in research studies involving the evaluation of investigational drugs or vaccines within 30 days of randomization.
- Suspected infection that would contraindicate steroid use (e.g. – Herpes)
- Known hypersensitivity to IVMP or one of its components or other contraindication to steroid therapy (e.g. - gastrointestinal bleeding).
- Preoperative use of mechanical circulatory support or active resuscitation at the time of proposed randomization.
- Inability to comply with study procedures.

4 Study Design

This is a multi-center, prospective, randomized, double blind, placebo controlled study of intraoperative MP in neonates undergoing CPB. Patients will be allocated to one of the 2 study groups in the ratio of 1:1. The randomization will be stratified by corrective vs. palliative surgery and by surgeon. The Primary Endpoint is a composite morbidity-mortality outcome, evaluated as intention to treat (ITT). Secondary endpoints include inotropic score, incidence of LCOS, fluid balance, ICU stay parameters, levels of inflammatory markers, safety parameters, and neurodevelopmental outcomes at 1 year. A two-sided test with a type I error rate of 0.05 will be used for all endpoints with no adjustments of the p-value for multiple testing.
It is expected that 190 patients (95 patients per treatment arm) will be need to be enrolled in the study to show a significant difference between groups. The annual amount of eligible & consenting patients at MUSC for this trial and a similar trial over 3 years was 35 cases/year. The annual amount of eligible patients at Children's Healthcare of Atlanta based on the surgical volume in 2012 would be 74 neonates. Anticipating a 70% consent rate would result in 50 cases a year. As such, this study would expect to reach the goal of 190 evaluable subjects in 4 years, within the 5 year proposed grant cycle.

4.1 Study Endpoints

4.1.1 Primary Endpoint

Composite Morbidity-Mortality Outcome

The composite morbidity-mortality outcome will be met if any of the following occur after surgery but before hospital discharge: death, cardiac arrest, extracorporeal membrane oxygenation, renal insufficiency (creatinine more than two times normal), hepatic insufficiency (aspartate aminotransferase or alanine aminotransferase more than two times normal > 36 hrs post-op), or lactic acidosis (>5mmol/L, that is increasing). This outcome is ideal because death rarely occurs in this population. Similar to others[107,114,115], we have found this endpoint to be highly associated with other important clinical outcomes in this population (Table 1). [117]

4.1.2 Secondary Endpoint

Compare the effects of MP to placebo on inotropic requirements, incidence of low cardiac output syndrome and fluid balance over the first 36 hours after surgery.

Compare the effects of MP to placebo on the duration of mechanical ventilation, ICU and hospital stay post cardiac surgery; and number of days alive and out of the hospital 90 days post cardiac surgery.

Compare the effects of MP to placebo on neurologic and developmental outcomes at 1 year.

Compare the effects of MP to placebo on the inflammatory response to cardiopulmonary bypass.

Compare the safety of MP to placebo following cardiac surgery.

5 STUDY TREATMENT REGIMEN

5.1 Premature Study Discontinuation

5.1.1 Premature Discontinuation of Study Treatment

Study treatment may be prematurely discontinued for any participant for any of the following reasons:

1. Study participation will be discontinued if a participant:
    a. Does not undergo cardiac surgery using cardiopulmonary bypass.
2. Study treatment may also be prematurely discontinued for any participant if the investigator or attending physician believes that the study treatment is no longer in the best interest of the participant.
3. Participants will be analyzed in the following groups
    a. Modified ITT sample – all randomized participants who underwent CPB.
    b. Safety sample – all randomized participants who underwent CPB and received study drug.
    c. Per protocol sample – All randomized participants who underwent CPB, received study drug, and had no major protocol deviations (i.e. open label steroids).

6 CRITERIA FOR PREMATURE TERMINATION OF THE STUDY

6.1 PARTICIPANT WITHDRAWAL CRITERIA

Participants may be prematurely terminated from the study for the following reasons:

1. The participant's parent(s)/LAR elects to withdraw consent from all future study activities, including follow-up.

Participants who prematurely terminate from this study due to withdrawal of consent will be included up until the point consent was withdrawn.

7 STUDY PROCEDURES

7.1 Enrollment and Screening Procedures

Other than the study drug regimen and schedule of measurements outlined below, subjects will receive all care and medications at the discretion of their attending physician.

Patients will be recruited from regular lines of referral of patients to pediatric cardiac surgical centers. Study subjects will consist of patients who are admitted for scheduled cardiac surgery requiring cardiopulmonary bypass. These patients are identified by their cardiac anatomy and age, which is known to their pediatric cardiologist and surgeon. For the purposes of this study, the principal investigator, co-investigator, or research coordinator will evaluate the eligibility of each patient scheduled for surgery. The Inclusion/Exclusion criteria will be evaluated and the patient recorded in a screening register. If the patient is considered to be a potential participant, then the entire study protocol will be discussed with the parent(s)/LAR. The PI, Co-I, and/or research coordinator will answer any and all questions, and the family will be allowed adequate time to consider patient participation. The consent form will be signed in accordance with all policies and regulations regarding consenting a minor patient. The original informed consent document will be retained within the subject's study file. The patient's parent(s)/LAR will give consent for this study as well as appropriate HIPAA consent. Copies of all consents will be given to the parent(s)/LAR and a note in the patient's chart will document consent.

7.1.1 Screening

The patient will be assessed for inclusion and exclusion criteria.

7.1.2 Enrollment

The subject's parent(s)/LAR will be approached prior to surgery by the PI, Co-I, and/or research coordinators who will introduce the study. If interested, the parent(s)/LAR will be provided a copy of the IFC to review and encouraged to ask questions. The consent will be reviewed in full and all questions will be answered. The consent can be signed on the day of surgery. A copy of the signed consent form will be given or mailed to the parent(s)/LAR after informed consent has been obtained. Enrollment will take place as close as possible to the day of surgery.

7.1.3 Randomization

This is a randomized placebo-controlled multicenter study of intraoperative MP in neonates undergoing CPB. The goal for subject randomization for this trial is to prevent potential selection biases, prevent serious imbalances in the distributions of important baseline prognostic factors and ensure the compatibility of the two subject groups. Based on knowledge from previous studies and clinical experiences, cardiac operation (corrective vs. palliative surgery) and surgeon are potential prognostic factors that may affect the primary outcome. Serious imbalances in the distribution of either of these two factors may reduce the compatibility of the study groups, and therefore affect the interpretation and the recipient of the trial results. Eligible subjects are randomized at 1:1 ratio to either the intraoperative methylprednisolone (MP) or placebo treatment group. See section 11 for more details. Randomization should occur as close as possible to scheduled surgery but may occur at any time prior to surgery.

7.1.4 Intraoperative Management

Study drug will be delivered to the operating room with the patient. The anesthesiologist will infuse the study drug intravenously over 30 minutes after central lines are placed and study labs have been drawn. The study drug will be started prior to skin incision. Intraoperative management, surgical and bypass techniques will not be standardized, but will be recorded in the CRFs.

7.1.5 Postoperative Management

All patients will be managed postoperatively in a dedicated pediatric cardiac intensive care unit by both a cardiac intensivist and cardiothoracic surgeon. Hemodynamic monitoring will minimally consist of ECG, invasive beat to beat arterial blood pressure, pulse oximetry, and noninvasive blood pressure. A urinary catheter will be used to assess total urinary output during the first 36 hours. hematology, biochemistry, blood gases, lactate and ionized calcium may be performed at the discretion of the attending physician. Cardiovascular medication will not be standardized, but will be recorded in the CRFs. To allow for the calculation of a vaso-active inotropic score [37, 107], the doses of inotropic medications will be recorded at admission to the ICU from the operating room and every 4-12 hours for 36 hours. The highest hourly inotropic score and vaso-active inotropic score over the first 36 hours postoperatively will also be recorded. Routine use of postoperative steroids will be discouraged.

7.1.6 Samples Collected

- **Blood collection** (5 mL/timepoint) will occur: prior to the administration of study drug (pre-CPB -baseline), Post-CPB, 4, 12, and 24 hours post-CPB resulting in a total collection of 25 mL over a 30 hour period.
- Two samples are collected in the operating room and are coincident with CPB priming.
- The 3 post-operative samples are taken with a minimum of an 8-hour sampling interval.

During the cooling and rewarming phases of CPB, routine clinical measurements of activated clotting time (ACT) are performed. The remaining discarded plasma may be retained for additional analysis.

The site of blood sampling will be a central venous line or arterial line that is placed as part of the routine clinical management of these patients, and no additional catheterization or venipuncture will be required.

- **Urine collection** will occur at 3 time points: immediately pre-CPB, 2 and 24 hours post-CPB.

Urine will be collected from a catheter that is placed as part of the routine clinical management. In the rare situation in which no urinary catheter is in place, no additional catheterization will occur and urine will not be collected. Data related to the level of circulating inflammatory and organ injury markers will be collected but run in a batch.

8 Study Drug Preparation and Administration

Study medication will be purchased, stored and tracked by Investigational Drug Services. When a patient is identified as eligible for the study and the parent(s)/LAR have given informed consent, the physician will provide an order to the investigational pharmacist. The investigational pharmacist will then assign the patient to the next available Subject ID on the corresponding list based on information on the surgeon and the surgery type, and prepare the study drug according to the treatment name provided on the randomization list.

Study medication:
Methylprednisolone: will be given at a dose of 30 mg/kg body weight and a concentration of 62.5 mg/cc mixed in a 3 mL syringe.

Placebo: Normal saline will be drawn up in an identical volume to that needed for active study drug.

The pharmacy will label the study medication with the special instructions to read:

"Send dose with patient to the OR to be administered IV over 30 minutes after central lines are placed and blood is drawn. Follow infusion with 3 mL Normal Saline Flush."

The study drug will be delivered in a blinded fashion to the anesthesiologist. The study drug will be administered intravenously over 30 minutes with the induction of anesthesia, after central lines have been placed and blood drawn, but prior to skin incision. The infusion will be followed by a 3 mL normal saline flush.

9 STUDY WINDOWS

Data collection should take place within the time limits below:

- In most cases, subject's parent(s)/LAR will be approached prior to surgery date, provided a copy of the consent and the informed consent discussion will begin.
- Consent signature can be obtained at any point prior to surgery.
- Randomization is considered enrollment in the study and can be performed at any point prior to surgery.
- Where possible, data should be entered within 72 hours of collection and/or availability.

10 SAFETY MONITORING

Adverse events that are classified as serious according to the definition set forth by the health authorities must be reported promptly to NHLBI, DSMB Chair, health authorities, principal investigators, and IRBs. This section defines the types of adverse events and outlines the procedures for appropriately collecting, grading, recording, and reporting them. Information in this section complies with *ICH Guideline E2A: Clinical Safety Data Management: Definitions and Standards for Expedited Reporting* and *ICH E6: Guideline for Good Clinical Practice,* and applies the standards set forth in the National Cancer Institute (NCI), *Common Terminology Criteria for Adverse Events version 3.0* (June 10, 2003).

10.1 Definitions

10.1.1 Adverse Event

The nature of the disease being study lends itself to a high morbidity and mortality rate. For this reason, Adverse Events (AEs) will be collected within the database. Data collection will include adverse events as:

- Mediastinitis or poor wound healing requiring surgical exploration
- CDC/NHSN defined blood stream infections, urinary tract infections, and pneumonia
- Hyperglycemia requiring insulin therapy within 36 hours of study drug
- Renal failure requiring renal replacement therapy within 48 hours of study drug

Regardless of the relationship of the adverse event to study drug, the event must be reported as an AE if it occurs anytime during the initial neonatal hospitalization for mediastinitis or poor wound healing requiring surgical exploration or infection. AEs will be reported to the site PI and the study PI as soon as they are found and within a week of discovery. All AEs will be reported to the DSMB at the semiannual meetings.

10.1.2 Serious Adverse Event

An SAE is defined as "any adverse event that suggests a significant hazard, contraindication, side effect, or precaution." The nature of the disease being study lends itself to a high morbidity and mortality rate. For this reason, SAEs will be collected within the database. Data collection will include events as:

- Death

Any death within the first year of life will be reported as an SAE. SAEs will be reported to the site PI and the study PI within 24 hours of discovering the event. All SAEs will be reported to the IRB within 72 hours of the PIs knowledge of the event. All SAEs will be reported to the local IRB/ DSMB chair within 72 hours of the PI's knowledge of the event.

10.1.3 Study Deviation

- Therapeutic Steroids

All surgeons and cardiac intensivists that staff our cardiac ICU have agreed to reserve additional steroid doses to the most critical scenarios. If given, it will be recorded in the CRF. Those patients will continue to be analyzed in the modified ITT sample and safety sample.

Those that receive steroids outside of the protocol will be considered a major protocol deviation and will be excluded from the per protocol sample.

10.2 Collection and Reporting of Events

10.2.1 Collection Procedure

Adverse events will be collected from the time the participant received study drug until the time the event is resolved or until 30 days after the participant completes study procedures, whichever comes first. Adverse events may be discovered through any of these methods:

- Observing the participant.
- Questioning the participant family; this should be done in an objective manner.
- Receiving an unsolicited complaint from the participant.
- An abnormal value or result from a clinical or laboratory evaluation (e.g., a radiograph, an ultrasound, or an electrocardiogram) can also indicate an adverse event.

10.2.2 Recording Procedure

Throughout the study the investigator will record all adverse events on the appropriate adverse event CRF. The investigator will treat participants experiencing adverse events appropriately and observe them at suitable intervals until their symptoms resolve or their status stabilizes.

10.2.3 SAE Grading and Attribution

The study site will grade the severity of adverse events according to the criteria set forth in the National Cancer Institute's *Common Terminology Criteria for Adverse Events Version 3.0* (published June 10, 2003). The grading system for this study will be defined as any adverse event meeting the following criteria:

Grade 3 = Severe and undesirable adverse event. (Marked limitation in activity, some assistance usually required; medical intervention/therapy required hospitalization possible.)
Grade 4 = Life-threatening or disabling adverse event.
Grade 5 = Death.

10.2.4 Reporting Procedure and Timeline

Any death within the first year of life will be reported as an SAE. SAEs will be reported to the site PI and the study PI and the NHLBI (sponsor) within 24 hours of discovering the event. All SAEs will be reported to the local IRB/ DSMB chair within 72 hours of the PI's knowledge of the event. In addition, the investigator must ensure that these events are entered on the SAE report form and the adverse event CRF and sent to the above-mentioned reviewers.

The DSMB Chair will make a determination if the DSMB must review immediately or not. Three way communication will continue until determinations are made for when and who will review it. All events will be in the routine DSMB report at a minimum. Ad Hoc DSMB review meetings can be called if necessary.

10.2.5 Data Safety Monitoring Plan

The Data Safety Monitoring Function will be done by an external Data Safety and Monitoring Board (DSMB) consisting of members at varying outside institutions using the Co-I Biostatistician on the project as the liaison to the group. The DSMB will consist of a minimum of 3 members. The DSMB will review the written protocol for this study prior to its initiation. The team will meet regularly (at least every 6 months) during the trial to review the data and any safety issues of the trial. In our summary statement to the DSMC, we will summarize the number of subjects enrolled in each group, along with descriptive statistics (e.g. frequencies, means, standard deviations, ranges) for all outcome variables.

Given the overall high prevalence of morbidity in this medically fragile study population, the PI will continually inform the DSMB of all SAE events in an expedited fashion. For an additional level of safety oversight, the PI will place enrollment on temporary hold when 1 year mortality exceeds 35.5% and the DSMB will be asked to provide an expedited data safety review, concluding with next step recommendations. This number is based on the PHN SVR trial demonstrating a 29% 1 year mortality.[47] For a sample size of 190 subjects assuming a 29% expected rate for death, the 95% confidence interval is 0.225 to 0.355.Interim analyses for efficacy and futility will be provided to the DSMB, as outlined in the protocol. The other investigators will remain blinded unless the DSMB determines that the study should be stopped. If there are safety concerns the DSMB may choose to meet more frequently or receive more frequent summary reports. A medical monitor will be available on site if needed.

We consider the planned interventions to be low risk but believe the DSMB will provide valuable input to the projects. The Principal Investigator will help to arrange the meetings and work with the statistician to develop the blinded reports. The statistician will provide any partially or fully unblinded reports to the DSMB.

As per HRPP 9.1 guidelines, each site will be responsible for reviewing research activities at the respective site. MUSC will remain the lead site, but IRB approval will occur at each site. Similarly adverse events will be reported to both sites simultaneously. There will be frequent contact between the 2 sites so that the lead center will know when each enrollment occurs. Amendments, continuing reviews, and protocol deviations will be conducted through the local IRB but immediately shared with the other participating IRB.

IRB - The study protocol, consent, and plans will be approved by the each institution's respective IRB prior to the conducting of the study and will be monitored during the study per local IRB procedures.

Data Processing - As case report forms are completed, the Research Coordinator will enter the data into the database and will keep such data entry up to date at least weekly. Any concerns or aberrations in protocol or procedures will be brought to the attention of the PI.

Data Security and Confidentiality - The database will be dedicated solely for use by this study protocol and will be password protected. Case report forms will be kept in a secure file within the Department of Pediatric Cardiology. Data files will be password protected. Personal identification information will only be kept by persons directly involved with the study and then only used for study protocol follow-up and safety monitoring.

10.2.6 Institutional Review Board Notification

The investigator will ensure the timely dissemination of SAE information based upon local IRB regulations and guidelines.

11 RANDOMIZATION, BLINDING, UNBLINDING

This is a randomized placebo-controlled multicenter study of intraoperative MP in neonates undergoing CPB. The goal for subject randomization for this trial is to prevent potential selection biases, prevent serious imbalances in the distributions of important baseline prognostic factors and ensure the compatibility of the two subject groups. Based on knowledge from previous studies and clinical experiences, cardiac operation (corrective vs. palliative surgery) and surgeon are potential prognostic factors that may affect the primary outcome. Serious imbalances in the distribution of either of these two factors may reduce the compatibility of the study groups, and therefore affect the interpretation and the recipient of the trial results. Eligible subjects are randomized at 1:1 ratio to either the intraoperative methylprednisolone (MP) or placebo treatment group.

11.1 Randomization

11.1.1 Randomization Code Development

The randomization scheme will be designed by the project biostatistician in collaboration with the investigational pharmacy. A permuted block randomization scheme stratified by cardiac operation and surgeon will be used so that imbalances between the two treatment group sizes within each stratum (cardiac operation by surgeon) will be contained. A list of randomization codes will be generated by the study statistician with information on stratum number, stratum name, a sequential ID, 4-digit subject randomization code in random order, treatment code (1 or 2), and treatment name (MP or placebo).

11.1.2 Randomization Code Management

A hard copy of the unblinded randomization code lists will be kept in the Central files at the Data Coordination Unit at MUSC.

11.2 Blinding

A hard copy of the site-specific unblinded randomization code list for each surgeon is sent to the site pharmacist, who is in charge of study drug kit preparation. This list contains all randomization codes for the surgeons in that site. This randomization list contains information on stratum number, stratum name, a sequential ID, the 4-digit subject randomization code, and column spaces for subject initials and date of randomization to be filled by the investigator during the randomization process.

The 4-digit randomization code is globally unique, and contains no information on treatment arm, time sequence of randomization, surgeon, and cardiac operation.
When randomized, the investigator will give the randomization code to the site pharmacist, who will then check the unblinded randomization code list and find out the treatment assignment associated with the randomization code. A study drug will be prepared accordingly and will be used by the investigator for study subject treatment.

11.3 Unblinding

When clinically justified by the treating physician of the study subject, treatment assignment for a specific subject can be unblinded. In this case, the study team member must first contact the study PI, Eric Graham and discuss the reason for unblinding. If it is determined that unblinding is necessary, then Dr. Graham will log the subject ID and reason for unblinding. The site will then be directed to contact the central pharmacy for unblinding. For each unblinding cases, a document will be created with detailed information on the site, surgeon, subject, reason for unblinding, the time, and the names of every investigator who is unblinded for the treatment assignment of the subject. An SAE form will be completed for each unblinded subject and the information will be forwarded to the DSMB chair in an expedited manner.

12 Statistical Considerations/Analytical Plan

12.1 Sample Size and Power Calculation For Primary Endpoint

Using data from the previous steroid study performed by Dr. Graham the incidence of a similar composite endpoint occurred in 33% of the subjects. This proposed study is powered to detect an absolute difference of 20% between groups. Enrollment of 190 patients (95 per treatment group) will be required to provide 80% power to detect this difference between groups, with an experiment wise alpha of 0.05 (two-sided), allowing for 1 interim look at the data to assess for superiority and futility. The power analysis was performed using Cytel's East software package (Cambridge, MA) and assumes an O'Brien Fleming alpha spending function.

12.2 Sample Size and Power Calculation for Secondary Endpoints

From the wide variety of Secondary Endpoints, a subset have available data to statistically assess whether or not they are adequately powered from the enrollment necessary to power the Primary Endpoint.

- ICU Length of Stay (LOS). Preliminary data suggest that ICU LOS data are highly skewed in this population, with 20% having ICU LOS > 10 days. We performed statistical simulations (with 5,000 replications) in which we assumed that 40% of subjects receiving placebo would have ICU LOS > 10 days but similar distribution within the ≤10 day and >10 day categories, and under such assumptions we found that having 95 subjects per group provided about 75% power to detect a difference of this magnitude.

- Maximum Inotropic Score Over 36 Hours. Graham [5] found maximum inotropic scores over the first 36 hours postoperatively of 14.4 ± 3.6 vs. 15.0 ± 3.9 for intraoperative MP alone vs. combined preoperative and operative MP. Assuming similar standard deviations in this study, 95 patients per arm with analyzable data will provide >95% power to detect a difference of ≥2 between groups.

- Neurodevelopmental Outcomes. The Pediatric Heart Network Single Ventricle Reconstruction Trial [47], evaluated neurodevelopmental outcomes at 12 months of age using the Bayley Scales of Infant Development. Their results revealed a mental scale raw score of 91.93 with a standard deviation of 7.25 in 172 infants undergoing staged palliation for hypoplastic left heart syndrome and related variants. Although dropout is unlikely prior to assessing the primary endpoint, it is more likely prior to the 12 month follow-up. Assuming a similar standard deviation and a 15% dropout rate, 81 patients per arm with analyzable data will provide 99% power to detect a difference of 5 points.

12.3 Subgroup Analyses

The modified ITT population, defined as the set of all patients randomized to treatment and underwent cardiopulmonary bypass, will be analyzed and presented for the Primary Endpoint. The same analyses will be conducted on the PP population, defined as those patients who are randomized, receive study drug as defined in the protocol, and have no major protocol violations preventing analysis (e.g. – post-op patients who receive steroids off-protocol). A two-sided test

with alpha of 0.05 will be used. The following subgroups will be analyzed for the Primary Endpoint:

Underlying diagnosis:	Palliated vs. Corrective procedure
Age:	≤ 7 days vs. >7 days
Body weight:	≤ 2.5 kg vs. >2.5 kg

12.4 Statistical Methods

Descriptive statistics will be used to characterize the two treatment groups with respect to demographics and baseline clinical characteristics. T-tests, chi-square tests, and non-parametric statistical tests will be used, as appropriate, to compare the groups at baseline. Any significant imbalances will be accounted for in the analyses using multivariable techniques. The primary intention-to-treat analysis comparing the composite outcome between treatment groups will be conducted using generalized linear model. The primary analysis will be adjusted for underlying diagnosis and surgeon. Logistic regression models will be used for the per-protocol analysis and for analyses of secondary categorical outcomes. For continuous variables such as change from baseline in vital signs, hematology, and clinical chemistry parameters, analysis of covariance (ANCOVA) will be utilized with the baseline value used as covariates. Unless otherwise stated, baseline is defined as the last observation before the administration of the study drug. Time to event data (duration of mechanical ventilation, length of ICU stay, and length of hospitalization) will be assessed via non-parametric log-rank tests.

Once half of the study subjects have been recruited and assessed, an interim analysis will also be conducted and presented to the DSMC, for which the p-value thresholds for stopping the study for superiority or futility will be 0.003 and 0.72, respectively. The p-value used at the end of the study with full enrollment will be 0.049, preserving an experiment-wise 2-sided alpha level of 0.05. These stopping thresholds are based on an Obrien-Fleming alpha spending function. All analyses will be conducted in SAS v9.2 (Cary, NC).

Multiple correlation analyses will be performed between the inflammatory markers and other continuous outcome measures, such as fluid balance, inotropic score and hemodynamic variables. An assessment will also be made of the association between the inflammatory markers and categorical variables, such as the composite morbidity-mortality outcome and its components. Analyzed safety variables will include all reported adverse events, the incidence of acute renal injury and postoperative creatinine, hyperglycemia and infection. All patients who are randomized, received study drug, and underwent cardiopulmonary bypass will be included in the safety analyses.

12.5 Study Endpoint Assessment

12.5.1 Primary Endpoint

Composite Morbidity-Mortality Outcome
The composite morbidity-mortality outcome will be met if any of the following occur after surgery but before hospital discharge: death, cardiac arrest, extracorporeal membrane oxygenation, renal insufficiency (creatinine more than two times normal), hepatic insufficiency (aspartate aminotransferase or alanine aminotransferase more than two times normal >36 hours

post-op), or lactic acidosis (>5mmol/L, that is increasing). This outcome is ideal because death rarely occurs in this population. Similar to others[107,114,115], we have found this endpoint to be highly associated with other important clinical outcomes in this population (Table 1). [117]

A secondary analysis will be performed on the incidence of the Composite Morbidity-Mortality Outcome at 30 days post cardiac surgery (regardless of hospital discharge status).

12.5.2 Secondary Endpoints

- CPB data (minutes)
  - duration of Deep Hypothermic Circulatory Arrest (DHCA) (if utilized)
  - total duration of CPB
  - duration of myocardial ischemia (cross clamp time)

- Inotropic Score and Vaso-active Inotropic Score
  Inotropic score is calculated by the equation:
  (dopamine+dobutamine) + (milrinone x10) +
  [(epinephrine+norepinephrine+isoproterenol)x100]. All drug doses are in micrograms/kg/min.

- Vasoactive Inotopic score is calculated by the equation:
  (dopamine+dobutamine)] + (milrinone x10) +
  [(epinephrine+norepinephrine+isoproterenol)x100] + (vasopressin x10). All drug doses are in micrograms/kg/min except vasopressin which is miliunits/kg/min. To convert vasopressin from units/kg/min to miliunits/kg/min multiply by 1,000 and inserted into the equation.

- LCOS – Assessed over the first 36 hours
  LCOS will be determined to be present if there are clinical signs and symptoms (e.g., tachycardia, oliguria, cold extremities) which required one or more of the following interventions during the initial 36 hours post admission from the operating room: mechanical circulatory support, escalation of existing pharmacological circulatory support to >100% over baseline, or initiation of new pharmacological circulatory support. Despite the inherent subjectivity of this definition, it imposes no additional risk to calculate and will be included as a secondary endpoint.

- Hemodynamic Parameters
  Vital Signs: Heart rate, systolic, diastolic and mean arterial pressures and pulse oximeter saturation
  Left and right atrial pressures when monitoring lines are present
  Cerebral and somatic (renal) near infrared spectroscopy (NIRS) when present

- Laboratory Parameters
  Hematology: complete blood count (CBC)
  Biochemistry: Na^+, K^+, Cl^-, HCO_3, BUN, creatinine, glucose, AST, ALT, alkaline phosphatase, total protein, albumin and total bilirubin.
  Arterial Blood Gas: PO2, PCO2, pH, HCO3, Lactate, Ionized Calcium

These laboratory tests are standard of care and not required by the study. When available they will be recorded at the time points described in Table 2.

- Acute Kidney Injury
  The Acute Kidney Injury Network's criteria for acute kidney injury will be applied.[109] Specifically, postoperative acute kidney injury will be defined as an increase in serum creatinine above the pre-operative level by either an absolute value of >0.3 mg/dL, a ≥ 50% increase or urine output below 0.5 mL/kg/hr over a 6 hour period.

- Other Parameters
  Duration mechanical ventilation (hours), ICU stay (hours), and hospitalization (days) Fluid intake (ml) and urine output (ml). In addition the number of days alive and out of the hospital at 90 days post cardiac surgery will be recorded.

- Bayley Scales of Infant Development
  The Bayley Scales of Infant Development (BSID-III is the current version) is a standard series of measurements used primarily to assess the motor (fine and gross), language (receptive and expressive), and cognitive development of infants and toddlers, ages 0-3. This measure consists of a series of developmental play tasks and takes between 45 - 60 minutes to administer. Raw scores of successfully completed items are converted to scale scores and to composite scores. These scores are used to determine the child's performance compared with norms taken from typically developing children of their age. This will be performed in conjunction with an evaluation from a specialist in Developmental Pediatrics at 12 months of age.

  The results of this evaluation will be made available to the parent(s)/LAR and pediatrician. Appropriate consultation with occupational, physical or speech therapist or follow up with Developmental Pediatrics will be made based on the specialist recommendations.

- Hemodynamics and Post-operative Course
  During the operative period, hemodynamic values will be recorded at time intervals coinciding with the blood collection. All measurements will be obtained from the catheters and monitors in place for the normal clinical conduct of the surgical procedure. In the postoperative period, the requirement for inotropic medications, duration of intubation and mechanical ventilation, duration of ICU and hospital stay, and fluid status will be documented from existing medical documentation. Any data obtained through the course of the study may be used for clinical management of the patient. Data related to the level of circulating biomarkers will be collected but run in batch at a core laboratory, therefore this will not be immediately available for direct patient care. All historical and demographic data will come from existing records.

- Quantitative Measures of Inflammatory Biomarkers
  Whole blood samples of 5 ml will be collected in a heparinized tube at critical peri/post-operative time points for the measurement of markers of oxidative stress, organ damage, cytokines, MMPs, and miRNAs. During the cooling and rewarming phases of CPB, routine clinical measurements of activated clotting time (ACT) are performed. The remaining

discarded plasma will be retained for additional analysis. This approach minimizes blood loss and provides additional CPB time points for analysis. Plasma will be isolated by centrifugation, decanted into aliquots and stored at -80°C until processed for immunoassays. All samples will be batched and run simultaneously to avoid potential laboratory assay variance. The list chosen here is not exhaustive, and would be expected to change as new information is discovered over the course of this study. The time course for the proposed measurements has been designed to minimize blood draws.

12.6 Study Data Collection

12.6.1 Table 2: Schedule of Measurements

	[a]PREOP	(c)T=1, 2 OR	PCICU	T=3 4 hrs	8 hrs	T=4 12 hr	T=5 24 hr	36 hr	D/C	30 days Post-op	90 days Post-op	1 Year Followup
**Windows**			Arrival	± 1 hr	± 1 hr	± 1 hr	± 1 hr	± 1 hr				12-14 Months
Informed Consent	X											
Inclusion/Exclusion	X											
Demographics	X											
Diagnosis Details		X										
Med/Surg History	X							X				
Randomization	X											
Vital Signs	X		X	X	X	X	X	X				
ABG, Ion Ca++, Lactate (e)	X		X	X	X	X	X	X				
Hematology (e)	X		X				X	X				
Biochemistry (e)	X		X				X	X				
Diuretics								X				
Biomarker		(b)XX		X		X	X					
Urine Sample		X	X				X					
Cardio-Pulmonary Bypass (CPB)		X										
Daily Inotropic Scores	X		X	X	X	X	X	X				
Highest Inotropic Score								X				
36 HR Assessment								X				
I/O (d)						X		X				
LCOS								X				
Duration of Ventilation									X			
Length of ICU & Hospital Stay									X			
Composite Outcome									X	X		
Days alive & out of hospital											X	
Bayley Scale												X
Study Completion												X
Adverse Events	X	X	X	X	X	X	X	X	X			X

(a) Last Values before Surgery
(b) 2 Biomarker Samples in OR regardless of time
(c) T= Time point of blood/urine sample for study
(d) I/O collected 1 day post-op
(e) These laboratory values are standard of care and will be recorded at these time points if obtained. They are not required

12.6.2 Medical and Surgical History

Medical history will be collected, including the existence of current signs and symptoms and clinical significance for each body system.

12.6.3 Study Completion

The percent of participants who complete the study, losses to follow-up, times to lost to follow-up, and reasons for discontinuation (e.g., adverse events) will be presented. Sample Size and Power Calculations

Some missing data are inevitable in any clinical study. The study will make every effort to minimize missing data through rigorous study procedures. However, when missing data occur, we will take steps in the analysis plan to understand any potential biases stemming from the missing data and to both describe and account for them. These methods include a comparison of those with and without missing data, documentation of sources of missing data, and evaluations of the pattern of missingness.

12.7 Sample Size and Power Calculation For Primary Endpoint

Using data from the previous steroid study performed by Dr. Graham the incidence of a similar composite endpoint occurred in 33% of the subjects. This proposed study is powered to detect an absolute difference of 20% between groups. Enrollment of 190 patients (95 per treatment group) will be required to provide 80% power to detect this difference between groups, with an experiment-wise alpha of 0.05 (two-sided), allowing for 1 interim look at the data to assess for superiority and futility. The power analysis was performed using Cytel's East software package (Cambridge, MA) and assumes an O'Brien Fleming alpha spending function.

12.8 Sample Size and Power Calculation for Secondary Endpoints

From the wide variety of Secondary Endpoints, a subset have available data to statistically assess whether or not they are adequately powered from the enrollment necessary to power the Primary Endpoint.

- ICU Length of Stay (LOS). Preliminary data suggest that ICU LOS data are highly skewed in this population, with 20% having ICU LOS > 10 days. We performed statistical simulations (with 5,000 replications) in which we assumed that 40% of subjects receiving placebo would have ICU LOS > 10 days but similar distribution within the ≤10 day and >10 day categories, and under such assumptions we found that having 95 subjects per group provided about 75% power to detect a difference of this magnitude.

- Maximum Inotropic Score Over 36 Hours. Graham [5] found maximum inotropic scores over the first 36 hours postoperatively of 14.4 ± 3.6 vs. 15.0 ± 3.9 for intraoperative MP alone vs. combined preoperative and operative MP. Assuming similar standard deviations in this study, 95 patients per arm with analyzable data will provide >95% power to detect a difference of ≥2 between groups.

- Neurodevelopmental Outcomes. The Pediatric Heart Network Single Ventricle Reconstruction Trial [47], evaluated neurodevelopmental outcomes at 12 months of age using

the Bayley Scales of Infant Development. Their results revealed a mental scale raw score of 91.93 with a standard deviation of 7.25 in 172 infants undergoing staged palliation for hypoplastic left heart syndrome and related variants. Although dropout is unlikely prior to assessing the primary endpoint, it is more likely prior to the 12 month follow-up. Assuming a similar standard deviation and a 15% dropout rate, 81 patients per arm with analyzable data will provide 99% power to detect a difference of 5 points.

12.9 Subgroup Analyses

The ITT population, defined as the set of all patients randomized to treatment, will be analyzed and presented for the Primary Endpoint. The same analyses will be conducted on the PP population, defined as those patients who are randomized, receive MP as defined in the protocol, and have no protocol violations preventing analysis (e.g. – post-op patients who receive steroids off-protocol). A two-sided test with alpha of 0.05 will be used. The following subgroups will be analyzed for the Primary Endpoint:

Underlying diagnosis:	Palliated vs. Corrective procedure
Age:	≤ 7 days vs. 8-31 days
Body weight:	≤ 2.5 kg vs. > 2.5 kg

12.10 Statistical Methods

Descriptive statistics will be used to characterize the two treatment groups with respect to demographics and baseline clinical characteristics. T-tests, chi-square tests, and non-parametric statistical tests will be used, as appropriate, to compare the groups at baseline. Any significant imbalances will be accounted for in the analyses using multivariable techniques. The primary intention-to-treat analysis comparing the composite outcome between treatment groups will be conducted using generalized linear model. The primary analysis will be adjusted for underlying diagnosis and surgeon. Logistic regression models will be used for the per-protocol analysis and for analyses of secondary categorical outcomes. For continuous variables such as change from baseline in vital signs, hematology, and clinical chemistry parameters, analysis of covariance (ANCOVA) will be utilized with the baseline value used as covariates. Unless otherwise stated, baseline is defined as the last observation before the administration of the study drug. Time to event data (duration of mechanical ventilation, length of ICU stay, and length of hospitalization) will be assessed via non-parametric log-rank tests.

Once half of the study subjects have been recruited and assessed, an interim analysis will also be conducted and presented to the DSMB, for which the p-value thresholds for stopping the study for superiority or futility will be 0.003 and 0.72, respectively. The p-value used at the end of the study with full enrollment will be 0.049, preserving an experimentwise 2-sided alpha level of 0.05. These stopping thresholds are based on an Obrien-Fleming alpha spending function. For an additional level of safety oversight, the PI will place enrollment on temporary hold if 1 year mortality exceeds 35.5% and the DSMB will be asked to provide an expedited data safety review, concluding with next step recommendations. All analyses will be conducted in SAS v9.2 (Cary, NC).

Multiple correlation analyses will be performed between the inflammatory markers and other continuous outcome measures, such as fluid balance, inotropic score and hemodynamic variables. An assessment will also be made of the association between the inflammatory markers and categorical variables, such as the composite morbidity-mortality outcome and its components. Analyzed safety variables will include all reported adverse events, the incidence of acute renal injury and postoperative creatinine, hyperglycemia and infection.

12.11 Reporting Deviations from Original Statistical Plan

The principal features of the study design and of the plan for statistical analysis of the data are outlined in this protocol and in the subsequent Statistical Analysis Plan (SAP). Any changes in these principal features will require a protocol or an SAP amendment, which would be subject to review by the independent DSMB, the study sponsor, and the health authorities. These changes will be described in the final report as appropriate.

13 Source Data

13.1 Identifying Source Data

The investigator is required to keep accurate records to ensure that the conduct of the study is fully documented. The results of all clinical and clinical laboratory evaluations will be maintained in the participant's medical records and the data will be transferred to clinical CRFs. Source data is any source from which the study data is first found. Source data is accompanied by the person making the entry with a date of the entry, or a time date stamp and the signature of the persons making the entry (either computer generated signatures or other means to track back the entry person).

13.2 Permitting Access to Source Data

The investigational site participating in this study will maintain the highest degree of confidentiality permitted for the clinical and research information obtained from the participants in this clinical trial. Medical and research records should be maintained at each site in the strictest confidence. However, as a part of the quality assurance and legal responsibilities of an investigation, the investigational site must permit authorized representatives of the sponsor(s) and health authorities to examine (and when required by applicable law, to copy) clinical records for the purpose of quality assurance reviews, audits, and evaluations of the study safety and progress. Unless required by the laws that permit copying of records, only the coded identity associated with documents or with other participant data may be copied (and all personally identifying information must be obscured). Authorized representatives as noted above are bound to maintain the strict confidentiality of medical and research information that is linked to identified individuals. The investigational site will normally be notified before auditing visits occur.

The database will be dedicated solely for use by this study protocol and will be password protected. Case report forms will be kept in a secure file within the Department of Pediatric Cardiology. Data files will be password protected. Personal identification information will only be kept by persons directly involved with the study and then only used for study protocol follow-up and safety monitoring.

14 QUALITY CONTROL AND QUALITY ASSURANCE

The investigator is required to keep accurate records to ensure that the conduct of the study is fully documented. All participating clinicians and project staff will receive specific training as necessary from local physician investigators and the overall project manager before assuming any study-specific roles. Quality control of the study will be maintained by conference calls that will include all study investigators and staff. These calls will review the status of the study at each site, difficulties encountered with study procedures and adverse events, enrollment figures, retention figures, and completion and accuracy of data for each site. Additional training will be provided as necessary on an individual or study-wide basis. Copies of the informed consent document will be retained in the local study file.

Each participating site will obtain human subjects approval. As all sites will have a review by the Human Subjects Protection Committee/IRB of all protocol versions, consent versions and adverse events reports. The DSMB will formally review enrollment and safety biannually and as needed.

Since some sites might include research assessments in their medical records, the confidentiality of this information will be specified in the consent forms. Any materials considered solely for research purposes will be kept in locked study files in research areas or password-protected databases and not included in medical records. Access to these data will be limited to study personnel. All investigators and project staff will maintain current human subjects training. No identifying information will be reported in papers or presentations; data will be reported in aggregate.

Data will be entered locally at each site into a Web-based database using the assigned study ID, participant initials and date of birth. This database will incorporate the latest technology in Web-based data entry, data validation, automatic user authentication and feedback, and security. Each site will maintain a separate file linking a patient's identity to his or her study ID.

The NHLBI is responsible for regularly reviewing the conduct of the trial, for verifying adherence to the protocol, and for confirming the completeness, consistency, and accuracy of all documented data.

14.1 Data Monitoring Plan

The data will be verified by a series of computerized edit checks, and all relevant data queries will be resolved regularly and the site investigators will audit 10% of the medical records reviewed to assess data quality. All discrepancies will be reviewed, and any resulting queries will be resolved with the investigator and amended in the database. All elements of data entry (i.e., time, date, verbatim text, and the name of the person performing the data entry) will be recorded in an electronic audit trail to allow all data changes in the database to be monitored and maintained in accordance with federal regulations.

Some missing data are inevitable in any clinical study. The study will make every effort to minimize missing data through rigorous study procedures.

15 Ethical Considerations and Compliance with Good Clinical Practice

15.1 Statement of Compliance

This clinical study will be conducted using good clinical practice (GCP), as delineated in *Guidance for Industry: E6 Good Clinical Practice Consolidated Guidance* [1], and according to the criteria specified in this study protocol. Before study initiation, the protocol and the informed consent documents will be reviewed and approved by an appropriate EC or IRB. Any amendments to the protocol or to the consent materials must also be approved before they are implemented.

15.2 Informed Consent and Assent

The informed consent form is a means of providing information about the trial to a prospective participant's parent(s)/LAR and allows for an informed decision about participation in the study. All participants (or parent(s)/LAR) must read, sign, and date a consent form before entering the study, taking study drug, or undergoing any study-specific procedures. Consent materials for participants who do not speak or read English must be translated into the participants' appropriate language.

The informed consent form must be revised whenever important new safety information is available, whenever the protocol is amended, and/or whenever any new information becomes available that may affect participation in the trial.

A copy of the informed consent will be given to a prospective parent(s)/LAR for review. The attending physician will review the consent and answer questions. The prospective subject's parent(s)/LAR will be told that being in the trial is voluntary and that he or she may withdraw from the study at any time, for any reason.

15.3 Cost to Participate

There is no cost to participate and each site will provide financial support for participants based upon their IRB's approved standard practice.

15.4 Privacy and Confidentiality

A participant's privacy and confidentiality will be respected throughout the study. Each participant will be assigned a sequential identification number, and these numbers rather than names will be used to collect, store, and report participant information. The log with the subject name and ID number will not be shared and will remain under lock and key at the individual sites. The data are housed in a secure data location accessible only by password and only study staff will be assigned access. Each site will adhere to HIPAA rules with no exceptions.

16 PUBLICATION POLICY

An objective of this study is to disseminate to the medical and lay communities new information so that evidence-based treatment strategies can be brought to bear. Our study leadership will develop and implement a dissemination plan that will include conventional publications in the medical literature, presentations, and notification of key news services for release of the trial results to the public. We propose the target audience to include, but not be limited to, pediatric cardiologists, congenital cardiac surgeons, pediatric cardiac anesthesiologists, pediatric cardiac intensivists, pediatric intensivists, general pediatricians, perfusionists, nurses, and other allied health specialists. National meetings of the AHA, Society of Thoracic Surgeons, American Association of Thoracic Surgeons, the ACC, the Pediatric Cardiac Intensive Care Society, and the American Society of Anesthesiologists are excellent venues for presenting clinical study results.

Each publication, press release, or other document about research supported by this NIH award will include an acknowledgement of NIH award support and a disclaimer such as "Research reported in this publication was supported by the National Heart, Lung, And Blood Institute of the National Institutes of Health under Award Number R02HL112968. The content is solely the responsibility of the authors and does not necessarily represent the official views of the National Institutes of Health." Prior to issuing a press release concerning the outcome of this research, the NIH will be notified to allow for coordination.

17 REFERENCES

(1) Thompson LD, McElhinney DB, Findlay P, Miller-Hance W, Chen MJ, Minami M, Petrossian E, Parry AJ, Reddy VM, Hanley FL. A prospective randomized study comparing volume-standardized modified and conventional ultrafiltration in pediatric cardiac surgery. J Thorac Cardiovasc Surg 2001 August;122(2):220-228.

(2) Turley K, Mavroudis C, Ebert PA. Repair of congenital cardiac lesions during the first week of life. Circulation 1982;66:I-214-I-219.

(3) Frantz S, Bauersachs J, Kelly RA. Innate immunity and the heart. Curr Pharm Des 2005;11(10):1279-1290.

(4) Tarnok A, Emmrich F. Immune consequences of pediatric and adult cardiovascular surgery: report of the 7th Leipzig workshop. Cytometry B Clin Cytom 2003 July;54(1):54-57.

(5) Graham EM, Atz AM, Butts RJ, Baker NL, Zyblewski SC, Deardorff RL, DeSantis SM, Reeves ST, Bradley SM, Spinale FG. Standardized preoperative corticosteroid treatment in neonates undergoing cardiac surgery: Results from a randomized trial. J Thorac Cardiovasc Surg 2011:142:1523-1529.

(6) McQuinn TC, Deardorff RL, Mukherjee R, Taylor AG, Graham EM, Atz AM, Forbus GA, DeSantis SM, Young JB, Stroud RE, Crawford FA, Bradley SM, Reeves ST, Spinale FG. Circulating matrix metalloproteinase levels after ventricular septal defect repair in infants. J Thorac Cardiovasc Surg 2010 December;140(6):1257-1265.

(7) Allen BS, Rahman S, Ilbawi MN, Kronon M, Bolling KS, Halldorsson AO, Feinberg H. Detrimental effects of cardiopulmonary bypass in cyanotic infants: preventing the reoxygenation injury. Ann Thorac Surg 1997;64:1381-1387.

(8) Alcaraz AJ, Sancho L, Manzano L, Esquivel F, Carillo A, Prieto A, Bernstein ED, Alvarez-Mon M. Newborn patients exhibit an unusual pattern of interleukin 10 and interferon gamma serum levels in response to cardiac surgery. J Thorac Cardiovasc Surg 2002;123:451-458.

(9) Seghaye MC. The clinical implications of the systemic inflammatory reaction related to cardiac operations in children. Cardiol Young 2003 June;13(3):228-239.

(10) Seghaye MC, Heyl W, Grabitz RG, Schumacher K, von BG, Rath W, Duchateau J. The production of pro- and anti-inflammatory cytokines in neonates assessed by stimulated whole cord blood culture and by plasma levels at birth. Biol Neonate 1998;73(4):220-227.

(11) Seghaye MC, Grabitz RG, Duchateau J, Busse S, Dabritz S, Koch D, Alzen G, Hornchen H, Messmer BJ, von BG. Inflammatory reaction and capillary leak syndrome related to cardiopulmonary bypass in neonates undergoing cardiac operations. J Thorac Cardiovasc Surg 1996 September;112(3):687-697.

(12) Seghaye MC, Duchateau J, Grabitz RG, Nitsch G, Marcus C, Messmer BJ, von BG. Complement, leukocytes, and leukocyte elastase in full-term neonates undergoing cardiac operation. J Thorac Cardiovasc Surg 1994 July;108(1):29-36.

(13) Ashraf S, Bhattacharya K, Tian Y, Watterson K. Cytokine and S100B levels in paediatric patients undergoing corrective cardiac surgery with or without total circulatory arrest. Eur J Cardiothorac Surg 1999 July;16(1):32-37.

(14) Alcaraz AJ, Manzano L, Sancho L, Vigil MD, Esquivel F, Maroto E, Reyes E, varez-Mon M. Different proinflammatory cytokine serum pattern in neonate patients undergoing open heart surgery. Relevance of IL-8. J Clin Immunol 2005 May;25(3):238-245.

(15) Stocker CF, Shekerdemian LS, visvanathan K, Skinner N, Brizard CP, Carlin JB, Horton SB, Penny DJ. Cardiopulmonary bypass elicits a prominent innate immune response in children with congenital heart disease. J Thorac Cardiovasc Surg 2004;127:1523-1525.

(16) Qing M, Schumacher K, Heise R, Woltje M, Vazquez-Jimenez JF, Richter T, rranda-Carrero M, Hess J, von BG, Seghaye MC. Intramyocardial synthesis of pro- and anti-inflammatory cytokines in infants with congenital cardiac defects. J Am Coll Cardiol 2003 June 18;41(12):2266-2274.

(17) Hovels-Gurich HH, Vazquez-Jimenez JF, Silvestri A, Schumacher K, Minkenberg R, Duchateau J, Messmer BJ, von BG, Seghaye MC. Production of proinflammatory cytokines and myocardial dysfunction after arterial switch operation in neonates with transposition of the great arteries. J Thorac Cardiovasc Surg 2002 October;124(4):811-820.

(18) Hovels-Gurich HH, Schumacher K, Vazquez-Jimenez JF, Qing M, Huffmeier U, Buding B, Messmer BJ, von BG, Seghaye MC. Cytokine balance in infants undergoing cardiac operation. Ann Thorac Surg 2002 February;73(2):601-608.

(19) Seghaye M, Duchateau J, Bruniaux J, Demontoux S, Bosson C, Serraf A, Lecronier G, Mokhfi E, Planche C. Interleukin-10 release related to cardiopulmonary bypass in infants undergoing cardiac operations. J Thorac Cardiovasc Surg 1996 March;111(3):545-553.

(20) Chaney MA. Corticosteroids and cardiopulmonary bypass: A review of clinical investigations. Chest 2002;121:921-931.

(21) Checchia PA, Bronicki RA, Costello JM, Nelson DP. Steroid use before pediatric cardiac operations using cardiopulmonary bypass: an international survey of 36 centers. Pediatr Crit Care Med 2005 July;6(4):441-444.

(22) Dickerson HA, Chang AC. Steroids and low cardiac output syndrome after cardiac surgery in children. Pediatr Crit Care Med 2005;6:495-496.

(23) Allen M, Sundararajan S, Pathan N, Burmester M, Macrae D. Anti-inflammatory modalities: their current use in pediatric cardiac surgery in the United Kingdom and Ireland. Pediatr Crit Care Med 2009 May;10(3):341-345.

(24) Hoffman TH, Wernovsky G, Atz AM, et al. Efficacy and safety of milrinone in preventing low cardiac output syndrome in infants and children after corrective surgery for congenital heart disease. Circulation 2003;107:996-1002.

(25) Buttgereit F, Saag KG, Cutolo M, da Silva JA, Bijlsma JW. The molecular basis for the effectiveness, toxicity, and resistance to glucocorticoids: focus on the treatment of rheumatoid arthritis. Scand J Rheumatol 2005;34:14-21.

(26) Tuckermann JP, Kleiman A, McPherson KG, Reichardt HM. Molecular mechanisms of glucocorticoids in the control of inflammation and lymphocyte apoptosis. Crit Rev Clin Lab Sci 2005;42:71-104.

(27) Bronicki RA, Backer CL, Baden HP, et al. Dexamethasone reduces the inflammatory response to cardiopulmonary bypass in children. Ann Thorac Surg 2000;69:1490-1495.

(28) Checchia PA, Backer CL, Bronicki RA, et al. Dexamethasone reduces the inflammatory response to cardiopulmonary bypass in children. Crit Care Med 2003;31:1742-1745.

(29) Schroeder VA, Pearl JM, Schwartz SM, Shanley TP, Manning PB, Nelson DP. Combined steroid treatment for congenital heart surgery improves oxygen delivery and reduces postbypass inflammatory mediator expression. Circulation 2003 June 10;107(22):2823-2828.

(30) Varan B, Tokel, K., Mercan S, et al. Systemic inflammatory response related to cardiopulmonary bypass and its modification by methyl prednisolone: High dose versus low dose. Pediatr Cardiol 2002;23:437-441.

(31) Mott AR, Fraser CD, Jr., Kusnoor AV, Giesecke NM, Reul GJ, Jr., Drescher KL, Watrin CH, Smith EO, Feltes TF. The effect of short-term prophylactic methylprednisolone on the incidence and severity of postpericardiotomy syndrome in children undergoing cardiac surgery with cardiopulmonary bypass. J Am Coll Cardiol 2001 May;37(6):1700-1706.

(32) Lindberg L, Forsell C, Jogi P, Olsson AK. Effects of dexamethasone on clinical course, C-reactive protein, S100B protein and von Willebrand factor antigen after paediatric cardiac surgery. Br J Anaesth 2003 June;90(6):728-732.

(33) Robertson-Malt S, Afrane B, El BM. Prophylactic steroids for pediatric open heart surgery. Cochrane Database Syst Rev 2007;(4):CD005550.

(34) Pasquali SK, Hall M, Li JS, Peterson ED, Jaggers J, Lodge AJ, Marino BS, Goodman DM, Shah SS. Corticosteroids and outcome in children undergoing congenital heart surgery: analysis of the

Pediatric Health Information Systems database. Circulation 2010 November 23;122(21):2123-2130.

(35) Newburger JW, Sleeper LA, McCrindle BW, Minich LL, Gersony W, Vetter VL, Atz AM, Li JS, Takahashi M, Baker AL, Colan SD, Mitchell PD, Klein GL, Sundel RP. Randomized trial of pulsed corticosteroid therapy for primary treatment of Kawasaki disease. N Engl J Med 2007 February 15;356(7):663-675.

(36) Parr GVS, Blackstone EH, Kirklin JW. Cardiac performance and mortality early after intracardiac surgery in infants and young children. Circulation 1975;51:867-874.

(37) Wernovsky G, Wypij D, Jonas RA, Mayer JEJr, Hanley FL, Hickey PR, Walsh AZ, Chang AC, Castaneda AR, Newburger JW, Wessel DL. Postoperative course and hemodynamic profile after the arterial switch operation in neonates and infants: a comparison of low-flow cardiopulmonary bypass and circulatory arres. Circulation 1995;92:2226-2235.

(38) DuPlessis AJ, Jonas RA, Wypij D, et al. Perioperative effects of alpha-stat versus pH-stat strategies for deep hypothermic cardiopulmonary bypass in infants. J Thorac Cardiovasc Surg 1997;114:991-1000.

(39) Pesonen EJ, Peltola KI, Korpela RE, Sairanen HI, Leijala MA, Raivio KO, Anderson SHM. Delayed impairment of cerebral oxygenation after deep hypothermic circulatory arrest in children. Ann Thorac Surg 1999;67:1765-1770.

(40) Antman EM. Medical management of the patient undergoing cardiac surgery. Heart Disease - A Textbook of Cardiovascular Medicine. 4th ed. Philadelphia, PA: W.B.Saunders Co., 1992:1677-1679.

(41) Burrows FA, Williams WG, Teoh KH, Wood AE, Burns J, Edmonds J, Barker GA, Trusler GA, Weisel RD. Myocardial performance after repair of congenital cardiac defects in infants and children. J Thorac Cardiovasc Surg 1988;96:548-556.

(42) Friedman WF. Congenital heart disease in infancy and childhood. Heart Disease - A Textbook of Cardiovascular Medicine. 4th ed. Philadelphia, PA: W.B.Saunders Co., 1992:894.

(43) Greeley WJ, Kern FH, Ungerleider RM, et al. The effect of hypothermic cardiopulmonary bypass and total circulatory arrest on cerebral metabolism in neonates, infants, and children. J Thorac Cardiovasc Surg 1992;101:783-794.

(44) Seghaye MC, Duchateau J, Grabitz RG, Faymonville ML, Messmer BJ, Buro-Rathsmann K, von Bemuth G. Complement activation during cardiopulmonary bypass in infants and children. J Thorac Cardiovasc Surg 1993;106:978-987.

(45) Charpie JR, Dekeon MK, Goldberg CS, Mosca RS, Bove EL, Kulik TJ. Serial blood lactate measurements predict early outcome after neonatal repair or palliation for complex congenital heart disease. J Thorac Cardiovasc Surg 2000;120:73-80.

(46) Lodge AJ, Chai PJ, Daggett CW, et al. Methyl-prednisolone reduces the inflammatory response to cardiopulmonary bypass in neonatal piglets: Timing of dose is important. J Thorac Cardiovasc Surg 1999;117:515-522.

(47) Ohye RG, Sleeper LA, Mahony L, Newburger JW, Pearson GD, Lu M, Goldberg CS, Tabbutt S, Frommelt PC, Ghanayem NS, Laussen PC, Rhodes JF, Lewis AB, Mital S, Ravishankar C, Williams IA, Dunbar-Masterson C, Atz AM, Colan S, Minich LL, Pizarro C, Kanter KR, Jaggers J, Jacobs JP, Krawczeski CD, Pike N, McCrindle BW, Virzi L, Gaynor JW. Comparison of shunt types in the Norwood procedure for single-ventricle lesions. N Engl J Med 2010 May 27;362(21):1980-1992.

(48) Hsia TY, McQuinn TC, Mukherjee R, Deardorff RL, Squires JE, Stroud RE, Crawford FA, Bradley SM, Reeves ST, Spinale FG. Effects of aprotinin or tranexamic acid on proteolytic/cytokine profiles in infants after cardiac surgery. Ann Thorac Surg 2010 June;89(6):1843-1852.

(49) Mann DL. Targeted anticytokine therapy in the failing heart. Am J Cardiol 2005;95(Suppl):9C-16C.

(50) Najm HK, Wallen WJ, Belanger MP, Williams WG, Coles JG, Van Arsdell GS, Black MD, Boutin C, Wittnich C. Does the degree of cyanosis affect myocardial adenosine triphosphate levels and function in children undergoing surgical procedures for congenital heart disease? J Thorac Cardiovasc Surg 2000;119:515-524.
(51) Burns SA, Newburger JW, Xiao M, Mayer JEJ, Walsh AZ, Neufeld EJ. Induction of interleukin-8 messenger RNA in heart and skeletal muscle during pediatric cardiopulmonary bypass. Circulation 1995;92:II-315-II-321.
(52) Haase E, Bigam DL, Nakonechny QB, Rayner D, Korbutt G, Cheung PY. Cardiac function, myocardial glutathione, and matrix metalloproteinase-2 levels in hypoxic newborn pigs reoxygenated by 21%, 50%, or 100% oxygen. Shock 2005;23:383-389.
(53) Galley HF, Macaulay GD, Webster NR. Matrix metalloproteinase-9, tissue inhibitor of metalloproteinase-1 and tumour necrosis factor alpha release during cardiopulmonary bypass. Anaesthesia 2002;57:549-662.
(54) Christen S, Finckh B, Lykkesfeldt J, Gessler P, Frese-Schaper M, Nielsen P, Schmid ER, Schmitt B. Oxidative stress precedes peak systemic inflammatory response in pediatric patients undergoing cardiopulmonary bypass operation. Free Radic Biol Med 2005;38:1323-1332.
(55) Meldrum DR, Partrick DA, Cleveland JC, Shenkar R, Meldrum KK, Raiesdana A, Ayala A, Brown JW, Harken AH. On-pump coronary artery bypass surgery activates human myocardial NF-kappaB and increases TNF-alpha in the heart. J Surg Res 2003;112:175-178.
(56) Tian B, Liu J, Bitterman PB, Bache RJ. Mechanisms of cytokine induced NO-mediated cardiac fibroblast apoptosis. Am J Physiol Heart Circ Physiol 2002 November;283(5):H1958-H1967.
(57) Baud V, Karin M. Signal transduction by tumor necrosis factor and its relatives. Trends Cell Biol 2001 September;11(9):372-377.
(58) Dempsey PW, Doyle SE, He JQ, Cheng G. The signaling adaptors and pathways activated by TNF superfamily. Cytokine Growth Factor Rev 2003 June;14(3-4):193-209.
(59) Hammer S, Fuchs AT, Rinker C, Daebritz S, Kozlik-Feldmann R, Netz H. Interleukin-6 and procalcitonin in serum of children undergoing cardiac surgery with cardiopulmonary bypass. Acta Cardiol 2004;59:624-629.
(60) Berdat PA, Eichenberger E, Ebell J, Pfammatter JP, Pavlovic M, Zobrist C, Gygaz E, Nydegger U, Carrel T. Elimination of proinflammatory cytokines in pediatric cardiac surgery: analysis of ultrafiltration method and filter type. J Thorac Cardiovasc Surg 2004;127:1688-1696.
(61) Gessler P, Pretre R, Hohl V, Rousson V, Fischer J, Dahinden C. CXC-chemokine stimulation of neutrophils correlates with plasma levels of myeloperoxidase and lactoferrin and contributes to clinical outcome after pediatric cardiac surgery. Shock 2004;22:513-520.
(62) Nian M, Lee P, Khaper N, Liu P. Inflammatory cytokines and postmyocardial infarction remodeling. Circ Res 2004;94:1543-1553.
(63) Mehlhorn U, Bloch W. Role of cardiopulmonary bypass and cardioplegic arrest in the regulation of cardiac nitric oxide synthase activity. J Thorac Cardiovasc Surg 2002 August;124(2):418-419.
(64) Siwik DA, Colucci WS. Regulation of matrix metalloproteinases by cytokines and reactive oxygen/nitrogen species in the myocardium. Heart Fail Rev 2004 January;9(1):43-51.
(65) Jensen E, Bengtsson A, Berggren H, Ekroth R, Andreasson S. Clinical variables and pro-inflammatory activation in paediatric heart surgery. Scand Cardiovasc J 2001 July;35(3):201-206.
(66) el-Barbary M, Khabar KS. Soluble tumor necrosis factor receptor p55 predicts cytokinemia and systemic inflammatory response after cardiopulmonary bypass. Crit Care Med 2002 August;30(8):1712-1716.
(67) Ozawa T, Yashihara K, Koyama N, et al. Clinical efficacy of heparin-bonded bypass circuits related to cytokine responses in children. Ann Thorac Surg 2000;69:584-590.
(68) Jensen E, Andreasson S, Bengtsson A, Berggren H, Ekroth R, Lindholm L, Ouchterlony J. Influence of two different perfusion systems on inflammatory response in pediatric heart surgery. Ann Thorac Surg 2003 March;75(3):919-925.

(69) Tarnok A, Hambsch J, Emmrich F, Sack U, van SJ, Bellinghausen W, Borte M, Schneider P. Complement activation, cytokines, and adhesion molecules in children undergoing cardiac surgery with or without cardiopulmonary bypass. Pediatr Cardiol 1999 March;20(2):113-125.
(70) Dittrich S, Aktuerk D, Seitz S, Mehwald P, Schulte-Monting J, Schlensak C, Kececioglu D. Effects of ultrafiltration and peritoneal dialysis on proinflammatory cytokines during cardiopulmonary bypass surgery in newborns and infants. Eur J Cardiothorac Surg 2004 June;25(6):935-940.
(71) Ashraf SS, Tian Y, Cowan D, Shaikh R, Parsloe M, Martin P, Watterson KG. Proinflammatory cytokine release during pediatric cardiopulmonary bypass: influence of centrifugal and roller pumps. J Cardiothorac Vasc Anesth 1997 October;11(6):718-722.
(72) Cao CM, Xia Q, Bruce IC, Shen YL, Ye ZG, Lin GH, Chen JZ, Li GR. Influence of interleukin-2 on Ca2+ handling in rat ventricular myocytes. J Mol Cell Cardiol 2003 December;35(12):1491-1503.
(73) Siwik DA, Chang DL, Colucci WS. Interleukin-1beta and tumor necrosis factor-alpha decrease collagen synthesis and increase matrix metalloproteinase activity in cardiac fibroblasts in vitro. Circ Res 2000 June 23;86(12):1259-1265.
(74) Peng J, Gurantz D, Tran V, Cowling RT, Greenberg BH. Tumor necrosis factor-alpha-induced AT1 receptor upregulation enhances angiotensin II-mediated cardiac fibroblast responses that favor fibrosis. Circ Res 2002 December 13;91(12):1119-1126.
(75) Parissis JT, Adamopoulos S, Venetsanou KF, Mentzikof DG, Karas SM, Kremastinos DT. Serum profiles of C-C chemokines in acute myocardial infarction: possible implication in postinfarction left ventricular remodeling. J Interferon Cytokine Res 2002 February;22(2):223-229.
(76) Shimpo H, Shimamoto A, Sawamura Y, Fujinaga K, Kanemitsu S, Onoda K, Takao M, Mitani Y, Yada I. Ultrafiltration of the priming blood before cardiopulmonary bypass attenuates inflammatory response and improves postoperative clinical course in pediatric patients. Shock 2001;16 Suppl 1:51-54.
(77) Tsuruda T, Costello-Boerrigter LC, Burnett JC, Jr. Matrix metalloproteinases: pathways of induction by bioactive molecules. Heart Fail Rev 2004 January;9(1):53-61.
(78) Wainwright CL. Matrix metalloproteinases, oxidative stress and the acute response to acute myocardial ischaemia and reperfusion. Curr Opin Pharmacol 2004 April;4(2):132-138.
(79) Giomarelli P, Scolletta S, Borrelli E, Biagioli B. Myocardial and lung injury after cardiopulmonary bypass: role of interleukin (IL)-10. Ann Thorac Surg 2003 July;76(1):117-123.
(80) Li JS, Sanders SP, Perry AE, Stinnett SS, Jaggers J, Bokesch P, Reynolds L, Nassar R, Anderson PA. Pharmacokinetics and safety of TP10, soluble complement receptor 1, in infants undergoing cardiopulmonary bypass. Am Heart J 2004;147:173-180.
(81) Journois D, Israel-Biet D, Pouard P, Rolland B, Silvester W, Vouhe P, Safran D. High-volume, zero-balanced hemofiltration to reduce delayed inflammatory response to cardiopulmonary bypass in children. Anesthesiology 1996 November;85(5):965-976.
(82) Kawamura T, Inada K, Nara N, Wakusawa R, Endo S. Influence of methylprednisolone on cytokine balance during cardiac surgery. Crit Care Med 1999;27:545-548.
(83) Lindsey ML. MMP induction and inhibition in myocardial infarction. Heart Fail Rev 2004;9:7-19.
(84) Mott JD, Werb Z. Regulation of matrix biology by matrix metalloproteinases. Curr Opin Cell Biol 2004;16:558-564.
(85) Schulz CG, Sawicki G, Lemke RP, Roeten BM, Schulz R, Cheung PY. MMP-2 and MMP-9 and their tissue inhibitors in the plasma of preterm and term neonates. Pediatr Res 2004;55:794-801.
(86) Sweet DG, Halliday HL, Warner JA. Airway remodelling in chronic lung disease of prematurity. Paediatric Respiratory Reviews 2002;3:140-146.
(87) Winkler MK, Foldes JK, Bunn RC, Fowlkes JL. Implications for matrix metalloproteinases as modulators of pediatric lung disease. Am J Physiol - Lung Cell Mol Physiol 2003;284:L557-L565.

(88) Pender SL, Braegger C, Gunther U, Monteleone G, Meuli M, Schuppan D, Macdonald TT. Matrix metalloproteinases in necrotising enterocolitis. Pediatr Res 2003;54:160-164.
(89) Borke W, Munkeby BH, Halvorsen B, Bjornland K, Tunheim SH, Borge GIA, Thaulow E, Saugstad OD. Increased myocardial matrix metalloproteinases in hypoxic newborn pigs during resuscitation: effects of oxygen and carbon dioxide. Eur J Clin Invest 2004;34:459-466.
(90) Cheung PY, Sawicki G, Salas E, Etches PC, Schulz R, Radomski MW. The mechanisms of platelet dysfunction during extracorporeal membrane oxygenation in critically ill neonates. Crit Care Med 2000;28:2584-2590.
(91) Garner JR, Stroud RE, Finklea L, Ikonomidis JS, Dorman BH, Spinale FG. The effects of leukocyte reduction on matrix metalloproteinase release in cardiopulmonary bypass. J Extra Corpor Technol 2004;36:185-190.
(92) Schneider S, Gunasinghe H, Sistino J, Blackwell M, Spinale F. Effects of leukocyte depletion filters on matrix metalloproteinase activation in an extracorporeal circulation circuit. J Extra Corpor Technol 2003 June;35(2):139-142.
(93) Lin TC, Li CY, Tsai CS, Ku CH, Wu CT, Wong CS, Ho ST. Neutrophil-mediated secretion and activation of matrix metalloproteinase-9 during cardiac surgery with cardiopulmonary bypass. Anesth Analg 2005 June;100(6):1554-1560.
(94) Mayers I, Hurst T, Puttagunta L, Radomski A, Mycyk T, Sawicki G, Johnson D, Radomski MW. Cardiac surgery increases the activity of matrix metalloproteinases and nitric oxide synthase in human hearts. J Thorac Cardiovasc Surg 2001 October;122(4):746-752.
(95) Joffs C, Gunasinghe HR, Multani MM, Dorman BH, Kratz JM, Cumbley AJ3, Crawford FAJ, Spinale FG. Cardiopulmonary bypass induces the synthesis and release of matrix metalloproteinases. Ann Thorac Surg 2001;71:1518-1523.
(96) Lalu MM, Pasini E, Schulze CJ, Ferrari-Vivaldi M, Ferrari-Vivaldi G, Bachetti T, Schulz R. Ischaemia-reperfusion injury activates matrix metalloproteinases in the human heart. Eur Heart J 2005 January;26(1):27-35.
(97) Mayers I, Hurst T, Radomski A, Johnson D, Fricker S, Bridger G, Cameron B, Darkes M, Radomski MW. Increased matrix metalloproteinase activity after canine cardiopulmonary bypass is suppressed by a nitric oxide scavenger. J Thorac Cardiovasc Surg 2003 March;125(3):661-668.
(98) Bradham WS, Moe G, Wendt KA, Scott AA, Konig A, Romanova M, Naik G, Spinale FG. TNF-alpha and myocardial matrix metalloproteinases in heart failure: relationship to LV remodeling. Am J Physiol Heart Circ Physiol 2002 April;282(4):H1288-H1295.
(99) Lindsey ML, Mann DL, Entman ML, Spinale FG. Extracellular matrix remodeling following myocardial injury. Ann Med 2003;35(5):316-326.
(100) Deschamps AM, Spinale FG. Disruptions and detours in the myocardial matrix highway and heart failure. Curr Heart Fail Rep 2005 March;2(1):10-17.
(101) Ren G, Dewald O, Frangogiannis NG. Inflammatory mechanisms in myocardial infarction. Curr Drug Targets Inflamm Allergy 2003 September;2(3):242-256.
(102) Gunasinghe SK, Ikonomidis J, Spinale FG. Contributory role of matrix metalloproteinases in cardiovascular remodeling. Curr Drug Targets Cardiovasc Haematol Disord 2001 December;1(2):75-91.
(103) Taylor KM, Bain W.H., Jones J.V., Walker M.S. The effect of hemodilution on plasma levels of cortisol and free cortisol. J Thorac Cardiovasc Surg 1976;72:57-61.
(104) Sayed D, Rane S, Abdellatif M. MicroRNAs challenge the status quo of therapeutic targeting. J Cardiovasc Transl Res 2009 March;2(1):100-107.
(105) Seok HY, Wang DZ. The emerging role of microRNAs as a therapeutic target for cardiovascular disease. BioDrugs 2010 June;24(3):147-155.
(106) Han M, Toli J, Abdellatif M. MicroRNAs in the cardiovascular system. Curr Opin Cardiol 2011 May;26(3):181-189.

(107) Gaies MG, Gurney JG, Yen AH, Napoli ML, Gajarski RJ, Ohye RG, Charpie JR, Hirsch JC. Vasoactive-inotropic score as a predictor of morbidity and mortality in infants after cardiopulmonary bypass. Pediatr Crit Care Med 2010 March;11(2):234-238.

(108) Kulik TJ, Moler FW, Palmisano JM, Custer JR, Mosca RS, Bove EL, Bartlett RH. Outcome-associated factors in pediatric patients treated with extracorporeal membrane oxygenator after cardiac surgery. Circulation 1996 November 1;94(9 Suppl):II63-II68.

(109) Mehta RL, Kellum JA, Shah SV, Molitoris BA, Ronco C, Warnock DG, Levin A. Acute Kidney Injury Network: report of an initiative to improve outcomes in acute kidney injury. Crit Care 2007;11(2):R31.

(110) Hoffman TM, Wessel DL, Chang AC, Kulik TL, Atz AM, Nelson DP, Spray TL, Cook AC, Bauer JA, Wernovsky G. Milrinone Usage in the Neonate Following Congenital Cardiac Surgery: Experience from the PRIMACORP Trial. Society for Pediatric Research E-PAS2007:617360.6 (abstract). 2007.
Ref Type: Abstract

(111) Yeh TF, Lin JL, Lin HC, et al. Outcomes at school age after postnatal dexamethasone therapy for lung disease of prematurity. N Engl J Med 2004;350:1304-1313.

(112) Langley SM, Chai PJ, Jaggers JJ, et al. Preoperative high dose methylprednisolone attenuates the cerebral response to deep hypothermic circulatory arrest. Eur J Cardiothorac Surg 2000;17:279-286.

(113) Butts RJ, Scheurer MA, Atz AM, Zyblewski SC, Hulsey TC, Bradley SM, Graham EM. Comparison of maximum vasoactive inotropic score and low cardiac output syndrome as markers of early post-operative outcomes after neonatal cardiac surgery. Pediatr Cardiol. 2012; 33:633-638.

(114) Moga MA, Manlhiot C, Marwali EM, McCrindle BW, Van Arsdell GS, Schwartz SM. Hyperglycemia after pediatric cardiac surgery: Impact of age and residual lesions. Crit Care Med 2011;39:266-272.

(115) McCrindle BW, Claizia N, Khaikin S, Coles J, Holtby H, Schwartz S, Scherer S, Caldarone CA, Van Arsdell G, Manlhiot C, Redington AN. Remote ischemic preconditioning in children undergoing surgery with cardiopulmonary bypass: A single centre double-blinded randomized trial. Circulation 2011;124:A11013.

(116) Graham EM, Atz AM, Gillis J, DeSantis SM, Haney AL, Deardorff RL, Uber WE, Reeves ST, McGowan FX, Bradley SM, Spinale FG. Differential effects of aprotinin and tranexamic acid on outcomes and cytokine profiles in neonates undergoing cardiac surgery. J Thorac Cardiovasc Surg. 2012;143:1069-1076.

(117) Butts RJ, Scheurer MA, Zyblewski SC, Wahlquist AE, Nieter PJ, Bradley SM, Atz AM, Graham EM. A composite outcome for neonatal cardiac surgery research. J Thorac Cardiovasc Surg. 2014; 147:428-423.

Appendix 1. Targeted /Planned Enrollment Table

TARGETED/PLANNED ENROLLMENT: Number of Subjects			
**Ethnic Category**	**Sex/Gender**		
	**Females**	**Males**	**Total**
Hispanic or Latino	6	6	12
Not Hispanic or Latino	78	100	178
**Ethnic Category: Total of All Subjects ***	84	106	190
**Racial Categories**			
American Indian/Alaska Native	0	0	0
Asian	1	1	2
Native Hawaiian or Other Pacific Islander	0	0	0
Black or African American	38	46	84
White	46	58	104
**Racial Categories: Total of All Subjects ***	84	106	190

* The "Ethnic Category: Total of All Subjects" must be equal to the "Racial Categories: Total of All Subjects."

Appendix 2. Schedule of Multiplexer Studies Table

Schedule of Multiplexer Studies: Inflammatory and Other Specialized Markers

Assay Target	Baseline (Pre-incision, prior to study drug)	Termination of CPB	4 hrs	12 hrs	24 hrs
Cytokines					
IL-1beta	**X**	**X**	**X**	**X**	**X**
IL-2	**X**	**X**	**X**	**X**	**X**
IL-6	**X**	**X**	**X**	**X**	**X**
IL-8	**X**	**X**	**X**	**X**	**X**
IL-10	**X**	**X**	**X**	**X**	**X**
MCP-1 (MCAF)	**X**	**X**	**X**	**X**	**X**
TNFalpha	**X**	**X**	**X**	**X**	**X**
Matrix Metalloproteinases					
MMP-1	**X**	**X**	**X**	**X**	**X**
MMP-2	**X**	**X**	**X**	**X**	**X**
MMP-3	**X**	**X**	**X**	**X**	**X**
MMP-8	**X**	**X**	**X**	**X**	**X**
MMP-9	**X**	**X**	**X**	**X**	**X**
MMP-13	**X**	**X**	**X**	**X**	**X**
**microRNAs**					
miR-21	**X**	**X**	**X**	**X**	**X**
miR-133	**X**	**X**	**X**	**X**	**X**
miR-199a	**X**	**X**	**X**	**X**	**X**
miR-210	**X**	**X**	**X**	**X**	**X**
miR-494	**X**	**X**	**X**	**X**	**X**
