# Supplementary material for: Association of Neurodevelopmental Outcomes With Environmental Exposure to Cyclohexanone During Neonatal Congenital Cardiac Operations: A Secondary Analysis of a Randomized Clinical Trial
Source: JAMA Netw Open. 2020 May 6;3(5):e204070. doi: 10.1001/jamanetworkopen.2020.4070 (PMC7203603; doi:10.1001/jamanetworkopen.2020.4070)
Supplement: Supplement 2. — eFigure 1. Concentrations of Cyclohexanone and Its Metabolites in Serum eTable. Unadjusted Differences in Neurodevelopmental Composite Scores per Interquartile Range Increase in Concentrations of Cyclohexanone and the Molar Sum of Its Metabolites eFigure 2. Perioperative Cyclohexanone Concentrations [file jamanetwopen-3-e204070-s002.pdf]

Supplementary Online Content

Everett AD, Buckley JP, Ellis G, et al. Association of neurodevelopmental outcomes with environmental exposure to cyclohexanone during neonatal congenital cardiac operations: a secondary analysis of a randomized clinical trial. *JAMA Netw Open*. 2020;3(5):e204070. doi:10.1001/jamanetworkopen.2020.4070

eFigure 1. Concentrations of Cyclohexanone and Its Metabolites in Serum

eTable. Unadjusted Differences in Neurodevelopmental Composite Scores per Interquartile Range Increase in Concentrations of Cyclohexanone and the Molar Sum of Its Metabolites

eFigure 2. Perioperative Cyclohexanone Concentrations

This supplementary material has been provided by the authors to give readers additional information about their work.

© 2020 Everett AD et al. *JAMA Network Open.*

eFigure 1. Concentrations of Cyclohexanone and Its Metabolites in Serum

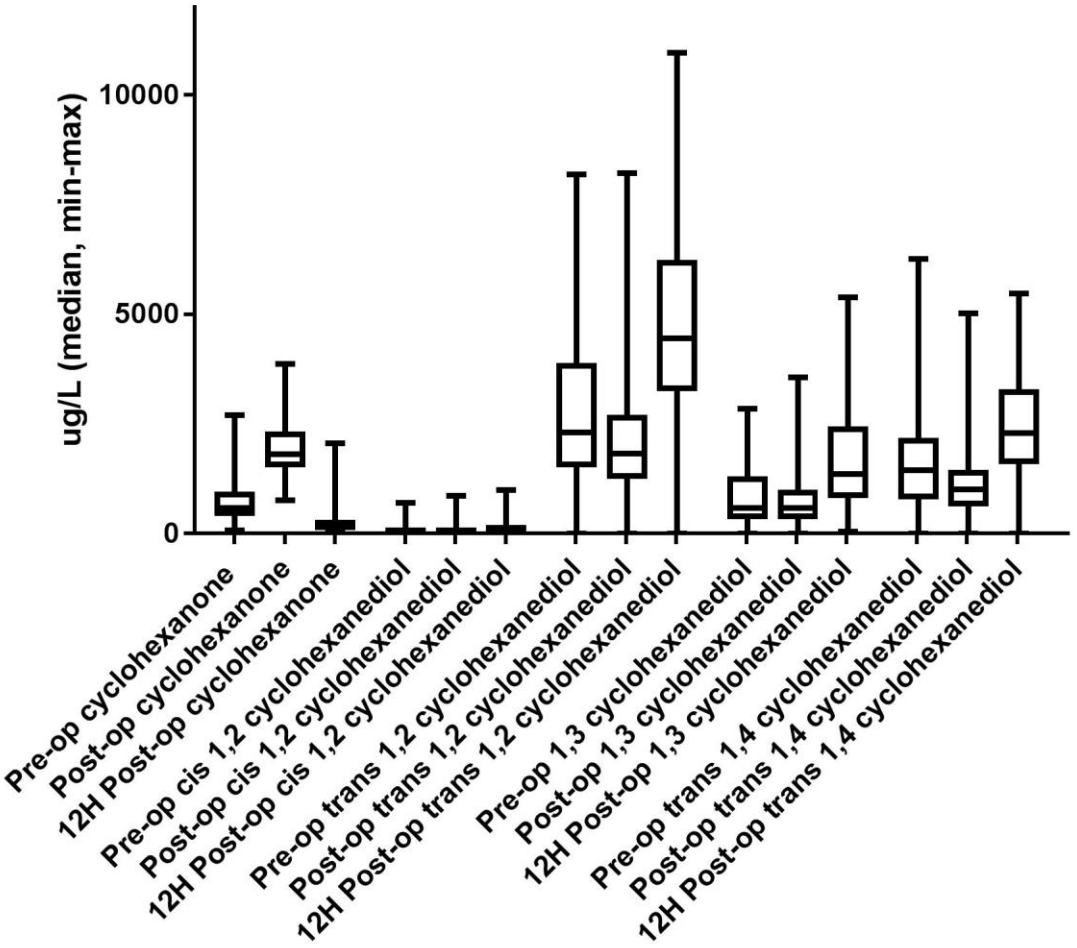

© 2020 Everett AD et al. *JAMA Network Open.*

eTable. Unadjusted Differences in Neurodevelopmental Composite Scores per Interquartile Range Increase in Concentrations of Cyclohexanone and the Molar Sum of Its Metabolites

Variable	N	Cognitive			Language			Motor		
		**β**	**95% CI**	**P**	**β**	**95% CI**	**P**	**β**	**95% CI**	**P**
Pre-op cyclohexanone	85	-2.02	-5.56, 1.52	0.267	-0.88	-4.10, 2.33	0.592	0.56	-4.01, 5.13	0.811
Pre-op Molar Sum Metabolites	82	-1.06	-3.91, 1.78	0.466	-0.52	-3.08, 2.04	0.691	-2.40	-6.01, 1.20	0.195
Post-operative cyclohexanone	85	-0.34	-3.56, 2.89	0.838	-0.02	-2.93, 2.89	0.987	-3.06	-7.14, 1.02	0.146
Post-operative Molar Sum Metabolites	85	**2.59**	**0.06, 5.12**	**0.048***	2.16	-0.13, 4.46	0.068	0.12	-3.20, 3.44	0.944
12H post-operative cyclohexanone	83	**-2.39**	**-4.10, -0.68**	**0.008***	**-2.02**	**-3.56, -0.47**	**0.012***	**-3.29**	**-5.46, -1.12**	**0.004***
12H post-operative Molar Sum Metabolites	80	2.65	-0.50, 5.81	0.104	**3.59**	**0.81, 6.38**	**0.013***	0.63	-3.47, 4.74	0.763
GM cyclohexanone	83	**-3.38**	**-6.28, -0.47**	**0.025***	**-2.69**	**-5.31, -0.07**	**0.048***	**-4.36**	**-8.08, -0.65**	**0.024***
GM Molar Sum Metabolites	77	2.98	-0.58, 6.55	0.105	3.13	-0.04, 6.30	0.057	-0.65	-5.26, 3.97	0.784

Abbreviation: GM, geometric mean
*P<0.05

© 2020 Everett AD et al. *JAMA Network Open.*

eFigure 2. Perioperative Cyclohexanone Concentrations

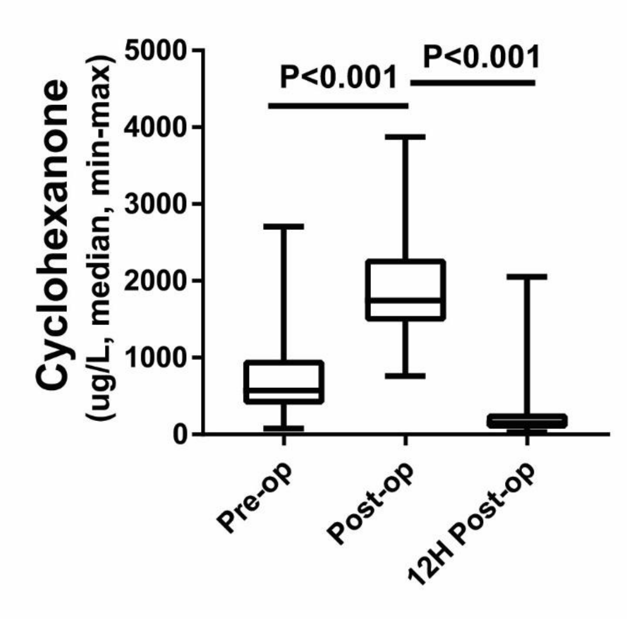

Cyclohexanone concentrations (ug/L median, min-max) at the Pre-op, immediately following CPB (Post-op) and 12 hours (H) post-op (12H Post-op). Post-op vs Pre-op and Post-op vs 12H post-op were significantly increased (P<0.001).

© 2020 Everett AD et al. *JAMA Network Open.*
