# Supplementary material for: Association of Neurodevelopmental Outcomes With Environmental Exposure to Cyclohexanone During Neonatal Congenital Cardiac Operations: A Secondary Analysis of a Randomized Clinical Trial
Source: JAMA Netw Open. 2020 May 6;3(5):e204070. doi: 10.1001/jamanetworkopen.2020.4070 (PMC7203603; doi:10.1001/jamanetworkopen.2020.4070)
Supplement: Supplement 3. — Data Sharing Statement [file jamanetwopen-3-e204070-s003.pdf]

Data Sharing Statement

Everett. Association of Neurodevelopmental Outcomes With Environmental Exposure to Cyclohexanone During Neonatal Congenital Cardiac Operations. *JAMA Netw Open*. Published May 06, 2020. 10.1001/jamanetworkopen.2020.4070

Data
Data available: Yes
Data types: Deidentified participant data
How to access data: grahamem@musc.edu
When available: beginning date: 01-01-2021, end date: 01-01-2022

Supporting Documents
Document types: None

Additional Information
Who can access the data: Researchers whose proposed use of the data is approved by the trial investigators
Types of analyses: Outcome analysis
Mechanisms of data availability: After approval of the proposal and a signed data use agreement
